# Biofabrication and biomanufacturing in Ireland and the UK

**DOI:** 10.1007/s42242-024-00316-z

**Published:** 2024-10-23

**Authors:** Jack F. Murphy, Martha Lavelle, Lisa Asciak, Ross Burdis, Hannah J. Levis, Cosimo Ligorio, Jamie McGuire, Marlene Polleres, Poppy O. Smith, Lucinda Tullie, Juan Uribe-Gomez, Biqiong Chen, Jonathan I. Dawson, Julien E. Gautrot, Nigel M. Hooper, Daniel J. Kelly, Vivian S. W. Li, Alvaro Mata, Abhay Pandit, James B. Phillips, Wenmiao Shu, Molly M. Stevens, Rachel L. Williams, James P. K. Armstrong, Yan Yan Shery Huang

**Affiliations:** 1 Department of Engineering, University of Cambridge, Cambridge, CB2 1PZ UK; 2 Department of Translational Health Sciences, Bristol Medical School, University of Bristol, Bristol, BS1 3NY UK; 3 Department of Biomedical Engineering, University of Strathclyde, Glasgow, G4 0NW UK; 4 Department of Materials, Institute of Biomedical Engineering, Imperial College London, London, SW7 2AZ UK; 5 Department of Bioengineering, Institute of Biomedical Engineering, Imperial College London, London, SW7 2AZ UK; 6 Department of Eye and Vision Science, Institute of Life Course and Medical Sciences, University of Liverpool, Liverpool, L7 8TX UK; 7 Biodiscovery Institute, University of Nottingham, Nottingham, NG7 2RD UK; 8 Department of Chemical and Environmental Engineering, University of Nottingham, Nottingham, NG7 2RD UK; 9 Centre for Human Development, Stem Cells and Regeneration, Human Development and Health, Faculty of Medicine, University of Southampton, Southampton, S016 6YD UK; 10 Division of Neuroscience, School of Biological Sciences, Faculty of Biology, Medicine and Health, University of Manchester, Manchester, M13 9PT UK; 11 UCL Centre for Nerve Engineering, Department of Pharmacology, UCL School of Pharmacy, University College London, London, WC1N 1AX UK; 12 Stem Cell and Cancer Biology Laboratory, The Francis Crick Institute, London, NW1 1AT UK; 13 CÚRAM, SFI Research Centre for Medical Devices, University of Galway, Galway, H91 W2TY Ireland; 14 School of Mechanical and Aerospace Engineering, Queen’s University Belfast, Belfast, BT9 5AH UK; 15 School of Engineering and Materials Science, Queen Mary University of London, London, E1 4NS UK; 16 Geoffrey Jefferson Brain Research Centre, Manchester Academic Health Science Centre, Northern Care Alliance and University of Manchester, Manchester, M13 9PL UK; 17 Trinity Centre for Biomedical Engineering, Trinity Biomedical Sciences Institute, Trinity College Dublin, Dublin 2, Ireland; 18 Department of Mechanical, Manufacturing and Biomedical Engineering, School of Engineering, Trinity College Dublin, Dublin 2, Ireland; 19 Advanced Materials and Bioengineering Research Centre (AMBER), Royal College of Surgeons in Ireland and Trinity College Dublin, Dublin 2, Ireland; 20 Department of Anatomy and Regenerative Medicine, Royal College of Surgeons in Ireland, Dublin, D02 H903 Ireland; 21 School of Pharmacy, University of Nottingham, Nottingham, NG7 2RD UK; 22 Department of Physiology, Anatomy and Genetics, Kavli Institute for Nanoscience Discovery, University of Oxford, Oxford, OX1 3QU UK; 23 Department of Engineering Science, Kavli Institute for Nanoscience Discovery, University of Oxford, Oxford, OX1 3QU UK

**Keywords:** Bioprinting, Drug delivery, Biomaterials, Tissue engineering, Sustainability, Biohybrid

## Abstract

As we navigate the transition from the Fourth to the Fifth Industrial Revolution, the emerging fields of biomanufacturing and biofabrication are transforming life sciences and healthcare. These sectors are benefiting from a synergy of synthetic and engineering biology, sustainable manufacturing, and integrated design principles. Advanced techniques such as 3D bioprinting, tissue engineering, directed assembly, and self-assembly are instrumental in creating biomimetic scaffolds, tissues, organoids, medical devices, and biohybrid systems. The field of biofabrication in the United Kingdom and Ireland is emerging as a pivotal force in bioscience and healthcare, propelled by cutting-edge research and development. Concentrating on the production of biologically functional products for use in drug delivery, in vitro models, and tissue engineering, research institutions across these regions are dedicated to innovating healthcare solutions that adhere to ethical standards while prioritising sustainability, affordability, and healthcare system benefits.

**Graphic abstract:**

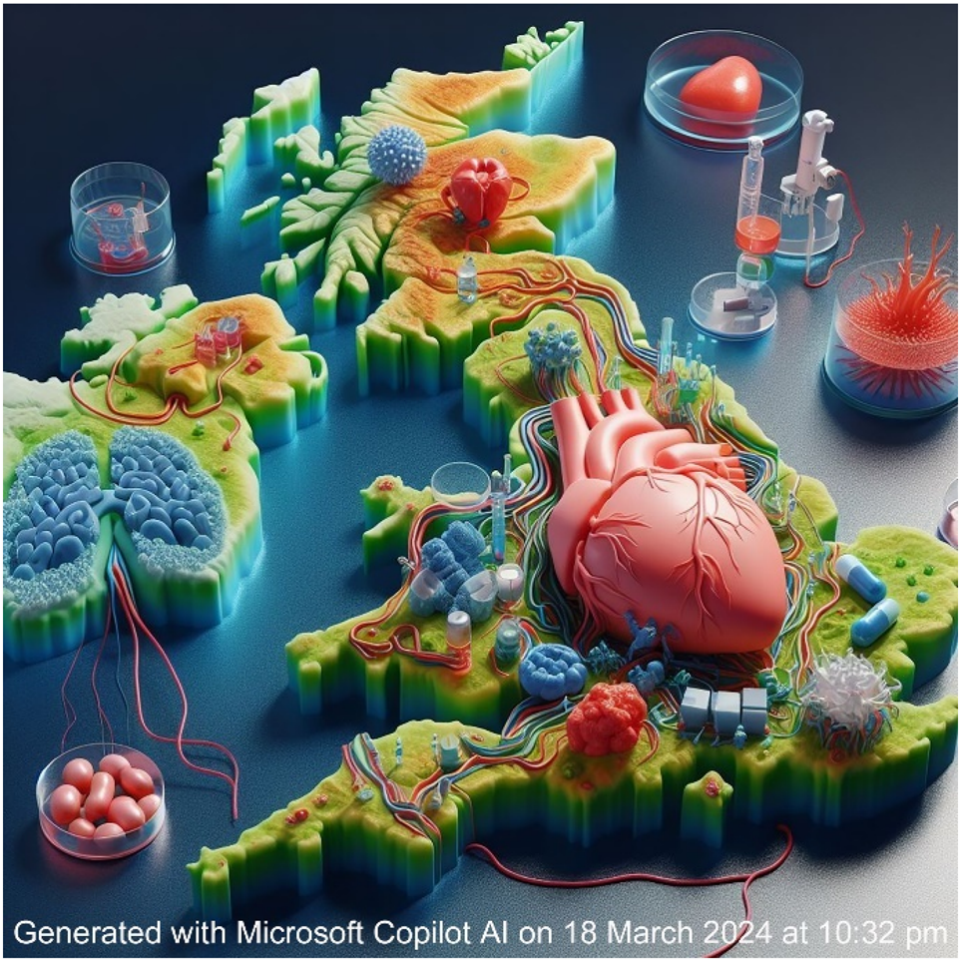

## Abstract (zh)

随着我们从第四次工业革命向第五次工业革命的过渡，生物制造领域的兴起正在改变生命科学和医疗保健。3D生物打印、组织工程、定向组装和自组装等先进技术在创建仿生支架、组织类器官、医疗设备和生物系统方面发挥着重要作用。英国和爱尔兰的生物制造领域成为生物科学和医疗保健的关键力量，得益于尖端的研究和发展。这些地区的研究专注于药物输送、体外模型, 类器官,和组织工程的生物功能产品，致力于创新符合伦理标准的医疗保健解决方案，同时支持环保和有效的医疗保健系统。

## Introduction

Human history has witnessed three major industrial revolutions, characterised by rapid technological advancements and global shifts in manufacturing techniques over relatively short time periods [1]. The First Industrial Revolution was coal-powered in the 1760s, followed by an oil and electricity technological revolution in the late nineteenth century (Second Industrial Revolution), and then a movement into the digital age in the late 1960s sparked by advances in electronics and nuclear energy (Third Industrial Revolution) [1]. Now, we are transitioning between the Fourth and Fifth Industrial Revolutions, which were precipitated by the rise of internet-connected devices and renewable energies. The Fourth Industrial Revolution, often referred to as Industry 4.0, saw a transition period from 2000 to 2010 and is characterised by the fusion of electronics, robotics, and biology [1], while Industry 5.0 seeks to implement these technologies in a sustainable manner that maximises their benefit to society. Integration of domains such as artificial intelligence, biotechnology, the Internet of Things, and three-dimensional (3D) printing is leading to rapid, global technological advancements that improve productivity and promote economic growth.

In particular, technological advances have transformed the life science and biomedical sectors. These innovations have brought significant economic and health benefits in the short- and medium-term, including a steady rise in life expectancy since the 1980s [2]. However, the long-term consequences cannot be ignored. For instance, the healthcare industry was responsible for twice as many carbon emissions as the aviation industry in 2019 [3, 4]. In addition, more than 100 million animals were used in research and testing globally in 2015 [5], and in 2022, 2.76 million scientific procedures were carried out on animals in the UK [6]. With fewer than 10% of drugs making it through this pipeline to clinical translation [7], these practices are unethical and unsustainable. The biomedical sector needs to act now to find alternative solutions because this problem will only continue growing as the industry scales up.

Biomanufacturing and biofabrication are rapidly growing areas that can be used to address these healthcare system shortcomings. This includes techniques such as 3D bioprinting, tissue engineering, and directed assembly, which are used to produce biologically-based products using living cells and organisms [8–11]. These techniques can be applied in vitro to model healthy and disease phenotypes, creating in vivo tissue and organ replacements, or fabricating sustainable biomaterials. A 2023 report by the World Health Organization considered 3D bioprinting as an emerging technology that could help solve global health challenges by increasing the supply of organs and tissues for drug screening and transplantation within the next 10 years [12]. Similarly, in 2023, the UK Department for Science, Innovation, and Technology recognised engineering biology as one of five critical technologies that will have a large impact on the UK economy by 2030 [13, 14]. This review seeks to collate the currently active research groups across Ireland and the UK using biofabrication techniques to translate lab-based research into positive socioeconomic impacts. This is not an in-depth review of all biofabrication and biomanufacturing processes; instead, it looks to highlight the ongoing research topics being pursued by these groups and see how they fit within the context of each other. Similar regional biofabrication reviews have explored the landscapes of 3D bioprinting in Israel [15] and biomanufacturing in Japan [16]. For in-depth reviews on specific biofabrication and 3D bioprinting techniques, the readers may refer to the following references [8, 17–21], including general bioprinting for tissue, organ, and organ-on-a-chip manufacturing [18, 20], 3D extrusion bioprinting [17], and using bioprinting to recapitulate the in vivo microenvironment [19, 21].

## Applications of biofabrication and biomanufacturing

Biofabrication is the process of constructing functional biological structures with living cells and biomaterials, whereas biomanufacturing employs biological systems for scalable production. The immediate impact of biofabrication and biomanufacturing emerges from developing and applying advanced biomaterials. Biomaterials have been around in various forms since antiquity; these biomaterials were initially chosen based on their mechanical and physical properties; if they could perform the mechanical function without being rejected by the immune system, then they were used [22]. This approach led to numerous “bio-inert” medical devices that have improved millions of lives and are still used today, such as prosthetics, stents, and dental implants [22]. More recently, the focus has been on “bio-active” biomaterials that can harness the host response as a resource to enhance tissue-biomaterial adhesion [23], stimulate local tissue regeneration [24], and release targeted therapeutic agents [25]. It is important to note that the fabrication methods used to create these biomaterials can change their mechanical and biochemical properties. For example, acid treatment unpacks the quaternary molecular folding of collagen extracted from tissue, making it thromboresistant and exposing peptide functional groups for cell attachment and signalling [26, 27].

The next step of biofabrication complexity is to directly integrate cells into these biomaterials. Traditional 2D culture studies have provided many insights into the inner workings of cell biology and uncovered many therapeutic pathways; however, they do not recapitulate the native microenvironment of cells in the human body [28]. For this reason, biofabricated materials are also used to support cell culture, providing substrates with tuneable stiffness, hydrophobicity, roughness, charge, and geometry that affect cell morphology and behaviour [29, 30]. Architecting biomaterials in 3D space is important for providing these biophysical and biochemical cues in a physiologically relevant manner. Biofabrication techniques such as 3D bioprinting [20, 31–33], directed assembly, and self-assembly can enable the fabrication of complex 3D biological structures. The shape of these structures can be physically engineered using geometric constraints [34]. Alternatively, they can be formed using remote manipulation methods such as acoustic [35] or magnetic [36] fields. Once formed, 3D aggregates often develop a necrotic core due to oxygen and nutrients being unable to perfuse through the bulk of the material [37]. For this reason, recent advances have sought to fabricate microvasculature in vitro [32, 38].

Biofabrication techniques have the potential to open up avenues for personalised medical solutions by using patient-derived cells to engineer a wide range of tissues [39], including bone [40], kidney [41], and myocardium models [42, 43]. This level of control over genetic background and tissue structure has allowed investigation of historically underrepresented demographics, including sex-specific differences in cardiac tissue [44, 45] and kidney [41] disease phenotypes. Although ethnic diversity has also been a target [46], greater diversity is still needed, with the vast majority of induced pluripotent stem cell (iPSC) lines being of European descent [47]. Effective use of the available biofabrication and biomanufacturing techniques can help reduce the pervasive gender and ethnicity biases that still present in many biomedical studies [48].

The applications of biofabrication and biomanufacturing are vast and not just limited to healthcare and the life sciences. The food, sensor, farming, construction, and fashion industries can use affordable bioprinting and other biofabrication technologies to improve their productivity and develop new products. In particular, small- and medium-sized enterprises, which make up 99% of all businesses in the European Union [49], do not have sufficient funds or expertise to develop their own techniques in house. For this reason, it is the responsibility of researchers in the field to develop low-cost and easy-to-deploy solutions that can be accessed by everybody. Without equitable and widespread distribution, the full benefit of these technologies will not be realised.

## Regional state-of-the-art technology in the early 2020s

**Fig. 1:**
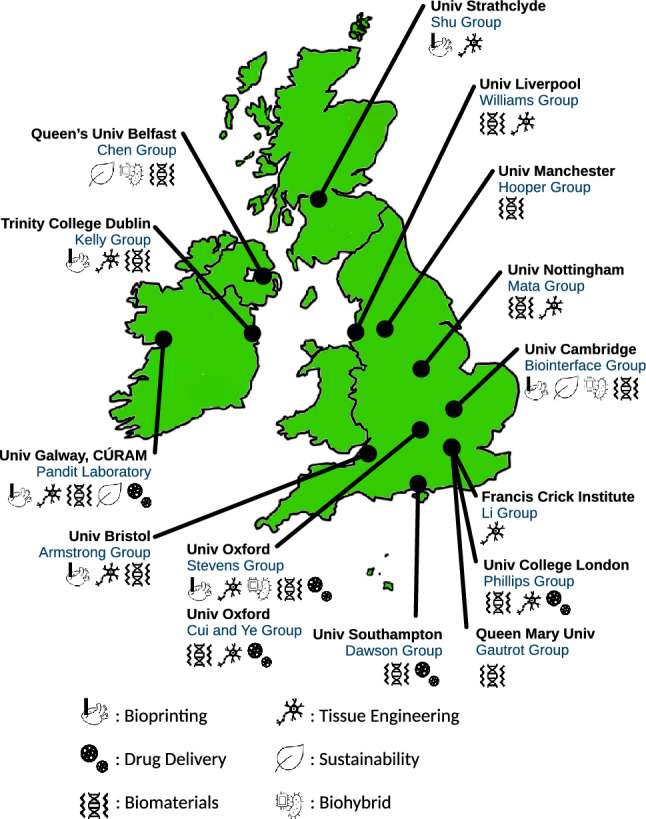
A non-exhaustive regional breakdown of biofabrication and biomanufacturing groups from around the UK and Ireland as of March 2024

This review surveys different research groups from across the UK and Ireland that are exploring the biomanufacturing and biofabrication sectors (Fig. 1 and Table 1). Research groups in this review are grouped by region to give a geographical breakdown of key biomanufacturing technologies being developed and utilised. With the size and rapidly evolving nature of the field, this review should not be considered an exhaustive list of biofabrication research being carried out in the UK and Ireland. Instead, it should be considered a snapshot of popular biofabrication sub-themes being explored on these isles.

**Table 1:** Summary of biofabrication research being investigated by groups in Ireland and the UK as of March 2024

Research group	Research summary	Applications
Pandit Laboratory, CÚRAM	Using glycosylation to improve scaffold biocompatibility, targeting specificity, and immunocompatibility	Enhancing efficacy and safety for scaffold-based drug delivery systems
Kelly Group	Bioprinting microtissues with a precise distribution of phenotypically distinct cell populations	Providing functional engineered grafts for damaged or diseased tissues
Chen Group	Synthesising and processing smart sustainable polymers and polymer nanocomposites for next-generation medical devices	Soft tissue engineering, drug delivery, wearables, soft robotics, wound healing
Mata Group	Leveraging biological organisation principles to create physiological structures and properties in vitro	Improving in vitro models of biological tissues
Williams Group	Fabricating biosynthetic corneal grafts using tuneable peptide hydrogels	Overcoming the donor shortage in cadaveric corneal grafts
Hooper Group	Mimicking the neurovascular unit with human pluripotent stem cells and collagen-based hydrogels	Investigating neurovascular dysfunction in Alzheimer’s disease
Gautrot Group	Design and patterning of polymer- and protein-based hydrogels and coatings for regenerative medicine	Stem cell delivery for soft tissue repair and nanotherapeutic design for RNA delivery
Li Group	Increasing the complexity, maturity, size of engineered intestinal tissues using 3D biofabrication techniques	Treating intestinal failure and modelling the gastro-intestinal tract
Phillips Group	Developing low-cost, high-throughput biomanufacturing techniques to produce living engineered neural tissues	Providing engineered neural tissue for nervous system repair
Biointerface Group	Developing 3D biofabrication techniques with a focus on sustainability and healthcare translatability	Sustainable modelling and monitoring of various living systems and system engineering biology
Stevens Group	Engineering nanotopographies for efficient cargo delivery and improving the versatility of bioprinting (perfusable channels, custom properties)	Improving drug delivery efficiency, enhancing the complexity of 3D bioprinted models
Cui and Ye Group	Harnessing tissue engineering and stem cell biology for regenerative medicine	Improving in vitro culture platforms and increasing efficacy of stem cell therapies
Dawson Group	Functionalising nanoclays to immobilise biomolecules and support cell growth, bone formation, and vascularisation	Improving drug delivery precision and efficacy
Armstrong Group	Developing biofabrication technologies that can be used to assemble complex tissues and organoids	Recreating structural complexity for in vitro modelling
Shu Group	Bioprinting human pluripotent stem cells, other cell types, and biomaterials without the need for complex machinery to produce functional, vascularised engineered tissues	Providing transplantable organs and realistic organ models, reducing animal testing

## Ireland

### Pandit Laboratory, CÚRAM, University of Galway

**Fig. 2:**
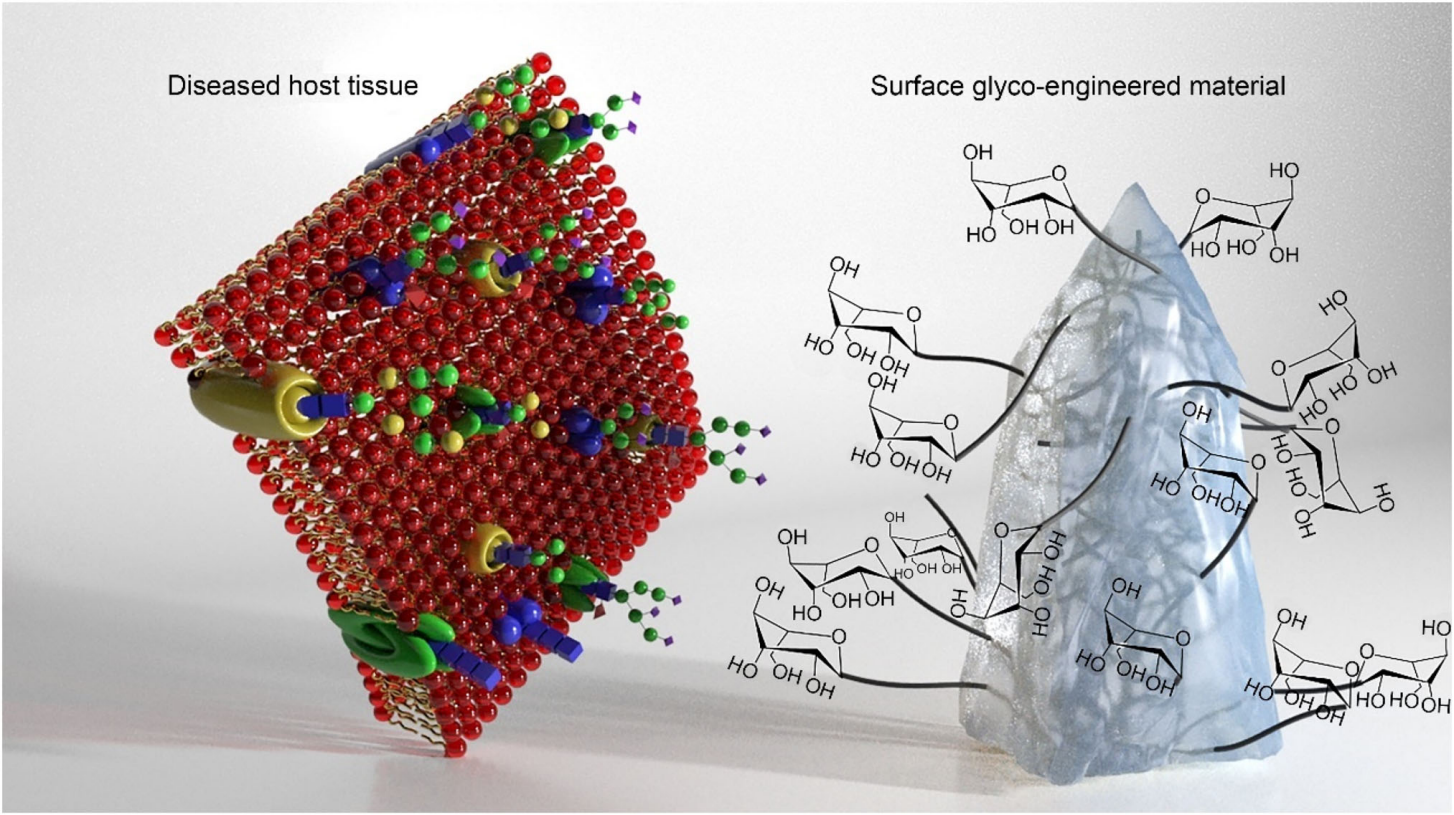
Next generation of implants: design of selective glyco-functionalised polymers will enable attachment of sugars to the end-groups of the polymer back bone to induce immunomodulation facilitating a desirable host response

Scaffold limitations in biomedical applications, such as drug delivery, are characterised by challenges related to biocompatibility, targeting specificity, and immunogenicity [50]. Glycosylation presents a potential solution to these limitations and is defined as the process of protein and lipid modification through the inclusion of glycans, which is a complex carbohydrate. This complex process involves the modification of macromolecular structures, genetic transcription, or protein translation and is subject to dynamic regulation through metabolic flux governed by glycotransferase enzymes, metabolites, and transporter proteins [51]. To improve materials for scaffolds and drug delivery, mimicking the glyco-profile of cells is critical [52]. Glycosylation can also enhance targeting specificity by modifying the surface properties of scaffolds through the attachment of specific sugar molecules, known as ligands or receptors, to the scaffold, allowing the targeting of specific cell types or tissues (Fig. 2). This is crucial for minimising side effects while directing drugs to specific body sites [53]. Furthermore, glycosylation can reduce the immunogenicity of unmodified scaffolds by masking antigenic epitopes on the scaffold surface, thereby decreasing the probability of an immune response [54]. Glycosylation offers a versatile method for addressing various limitations associated with scaffold-based drug delivery systems and enhancing the efficacy and safety profiles of drug delivery systems. However, it is crucial to recognise that the specific effects of glycosylation can vary depending on factors such as the type of scaffold, the nature of the attached glycans, and the intended application. During the past decade, the Pandit Laboratory has been exploring and developing novel glycosylated materials and establishing different synthetic routes for sustainable glyco-chemistry, such as collagen carbohydrate-functionalised hydrogels as neural glyco-environment modulators and glyco-modulators of extracellular matrix (ECM) inspired materials to target neuroinflammation. Future research on glycan modification will likely focus on incorporating glycan molecules into synthetic and natural polymers by controlling glyco-substitution and applying them as immunomodulators.

### Kelly Group, Trinity College Dublin

Advances in additive manufacturing and bioprinting have underpinned numerous important developments in the field of musculoskeletal tissue engineering. Early pioneering work in the field demonstrated the possibility of engineering geometrically complex tissues like the meniscus [55] or using printing to spatially pattern growth factors to direct bone or muscle formation [56]. More recently, the use of support baths has enabled bioprinting at much finer spatial resolutions with a diverse range of bioinks [57], while novel biofabrication approaches have helped to address important challenges such as dramatically reducing printing time [58], engineering of anisotropic tissues [59], vascularisation [38] or providing reinforcement to generate tissues with biomimetic mechanical properties [60]. Prof. Daniel Kelly at Trinity College Dublin and others in the field have also developed tissue-specific bioinks capable of supporting stem cell differentiation towards specialised cell types [61–64]. In spite of this, integrating such complexity into engineered tissues does not yet guarantee success in vivo. For example, Prof. Kelly’s group has demonstrated that bioprinted osteochondral implants, consisting of cell-laden bioinks reinforced by printed polymeric fibres and matured in vitro prior to implantation, do not consistently promote hyaline cartilage regeneration in large animal models of damaged synovial joints [65].

**Fig. 3:**
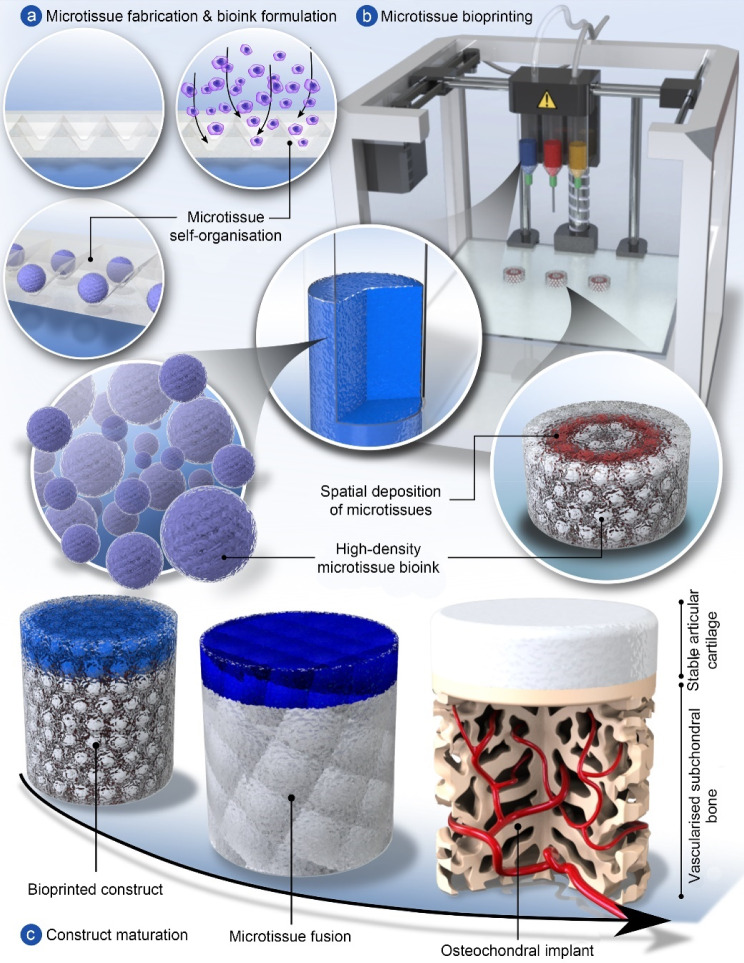
A potential biofabrication pathway for engineering complex osteochondral grafts. **a** Microtissues are biofabricated in a high-throughput fashion using micromoulding, whereby single cells self-organise into spherical cellular aggregates. Phenotypically distinct microtissues can be generated by using different cell types or by exposing aggregated stem cells to tissue-specific culture conditions. **b** Once formed, microtissue laden bioinks are deposited via bioprinting to create spatially organised constructs. **c** Following bioprinting, adjacent microtissues fuse to form a single macrotissue which is matured in vitro to create tissue rudiments for articular cartilage and subchondral bone. Once implanted, the precursor tissues continue to mature, mirroring the native postnatal osteochondral developmental program, resulting in the formation of a functional osteochondral unit and regeneration of the joint surface

A fundamental limitation of current approaches in biofabrication and bioprinting of tissues and organs is an inability to generate constructs with truly biomimetic composition, structure and function. This is perhaps unsurprising, as many tissues and organs continue to mature postnatally, often taking many years to attain the compositional and structural complexity that is integral to their function [66]. A potential solution to this challenge is to engineer tissues of intermediate complexity that are more representative of an earlier stage of development, using bioprinting not only to generate such constructs but also to provide them with guiding structures and biochemical cues that support their maturation into fully functional tissues or organs in vivo [67]. Towards this goal, Prof. Kelly has demonstrated that inkjet bioprinting can be used to deposit defined numbers of progenitor cells into additively manufactured scaffolds, wherein they form cellular aggregates that subsequently generate cartilage that recapitulates the complexity of skeletally immature native tissue in response to the physical cues provided by the scaffolding material [68–70]. Alternatively, it is possible to pre-engineer numerous microtissues or organoids and combine them with 3D printed structures that function to guide their fusion and remodelling into scaled-up grafts. In Prof. Kelly’s group, they have used such an approach to engineer living osteochondral grafts capable of directing joint regeneration in large animal pre-clinical models [71]. More biomimetic tissues can be engineered by using enzymes to accelerate the remodelling of such microtissues into structurally organised macrotissues [72]. Prof. Kelly’s group is currently focusing on using bioprinting tools to control the precise spatial deposition of phenotypically distinct microtissues, which can then be matured in vitro into patient-specific grafts for targeted clinical applications, e.g., osteochondral defect regeneration, see Fig. 3. It is their hope that such biofabrication and bioprinting approaches will enable the engineering of grafts with sufficient complexity to continue their development into fully functional tissues following their implantation into damaged or diseased regions of the body.

## Northern Ireland

### Chen Group, Queen’s University Belfast

Polymers and polymer nanocomposites are key materials in biofabrication and biomanufacturing. However, the vast majority of polymers are still produced from fossil fuels, which are not renewable. To help tackle sustainability challenges and develop innovative biomaterials for next-generation smart medical devices, Prof. Biqiong Chen and co-workers at Queen’s University Belfast are synthesising and processing smart, sustainable polymers and polymer nanocomposites for a variety of biomedical and healthcare applications.

It has been shown that biocompatible elastomers with controllable mechanical properties, biodegradation kinetics, and swelling behaviour can be synthesised and can subsequently be manufactured into biomimetic porous structures by lyophilisation-induced controlled phase separation (random, aligned, multilayered) for different types of soft tissue engineering (e.g., adipose, ligament, oral mucosal, cartilage) and wound healing [73–76]. Polymer nanocomposite hydrogels with extraordinary mechanical properties (e.g., tough, super-elastic with minimal hysteresis) have been fabricated for tissue repair and fluidic devices [77, 78]. Some of these polymers and polymer nanocomposites show stimuli responses (e.g., pH [76, 78], water [79], temperature [73], magnetic [80], electric [80, 81]) and demonstrate potential in pH-sensitive drug delivery [76], sustained and controlled drug delivery [73], transdermal drug delivery (in the form of microneedles) [80, 81], minimally invasive medical devices (through water-responsive shape memory effects) [79], actuation [73], and soft robotics.

Biobased thermoplastic polymers have been sustainably synthesised using monomers derived from plant oils [82, 83], which can be manufactured into healthcare products such as medical tubing, packaging, and medical equipment using polymer processing technologies, including extrusion and injection moulding. Biobased, self-healing thermoplastic elastomers have been used as substrates to develop stretchable/flexible electronics, sensors, e-skins, and wearable technologies through different printing technologies as well as smart coating and textiles [83, 84].

**Fig. 4:**
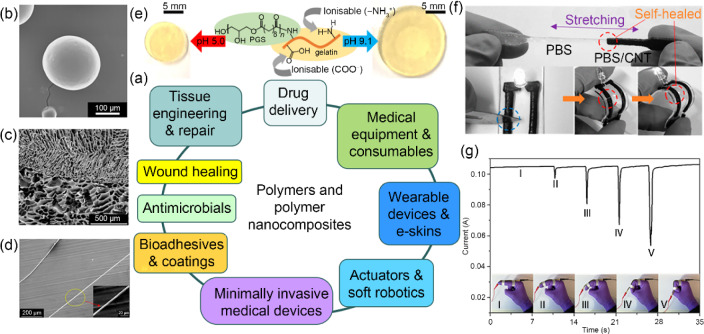
Applications of polymers and polymer nanocomposites in biomedical engineering and healthcare. **a** Diagram showing some important applications of polymers and polymer nanocomposites: **b** microspheres for drug delivery and injection therapy (reproduced from Ref. [73], Copyright 2015, with permission from The Royal Society of Chemistry), **c** bilayered porous scaffolds for tissue engineering (reproduced from Ref. [75], Copyright 2021, with permission from the authors, licensed under CC BY 4.0), **d** hydrogel microfibres (reproduced from Ref. [77], Copyright 2017, with permission from the American Chemical Society), **e** a pH-sensitive polymer (reproduced from Ref. [76], Copyright 2018, with permission from The Royal Society of Chemistry), **f** a self-healing polymer nanocomposite as a flexible electronic device (reproduced from Ref. [85], Copyright 2016, with permission from the American Chemical Society), and **g** a strain sensor for monitoring joint movements (reproduced from Ref. [86], Copyright 2017, with permission from the American Chemical Society)

These smart, sustainable polymers and polymer nanocomposites are versatile in biofabrication and biomanufacturing; they can be manufactured into different forms (e.g., bulk, films, foams, fibres, microspheres), shapes, and dimensions for various biomedical and healthcare applications. By controlling the molecular design and structure, the properties and functionality of polymers and polymer nanocomposites may be manipulated, which provides a solid basis for developing sustainable, high-performance, and personalised medical products. Figure 4 shows a summary of some important applications of polymers and polymer nanocomposites in biomedical engineering and healthcare [73, 75, 76, 85, 86].

## East Midlands, England

### Mata Group, University of Nottingham

Regenerative medicine and tissue engineering hold great promise for the restoration of injured and diseased tissues and organs [87]. To achieve these goals, it is critical to develop technologies that enable the fabrication of structures exhibiting the complexity and functionality of human tissues. Emerging fields such as precision medicine, which aims to enable personalised therapeutic screening, also rely heavily on the capacity to emulate features of biological tissues [88]. The re-creation of the structure and function of such living systems will require new ways of thinking and manufacturing paradigms [89].

**Fig. 5:**
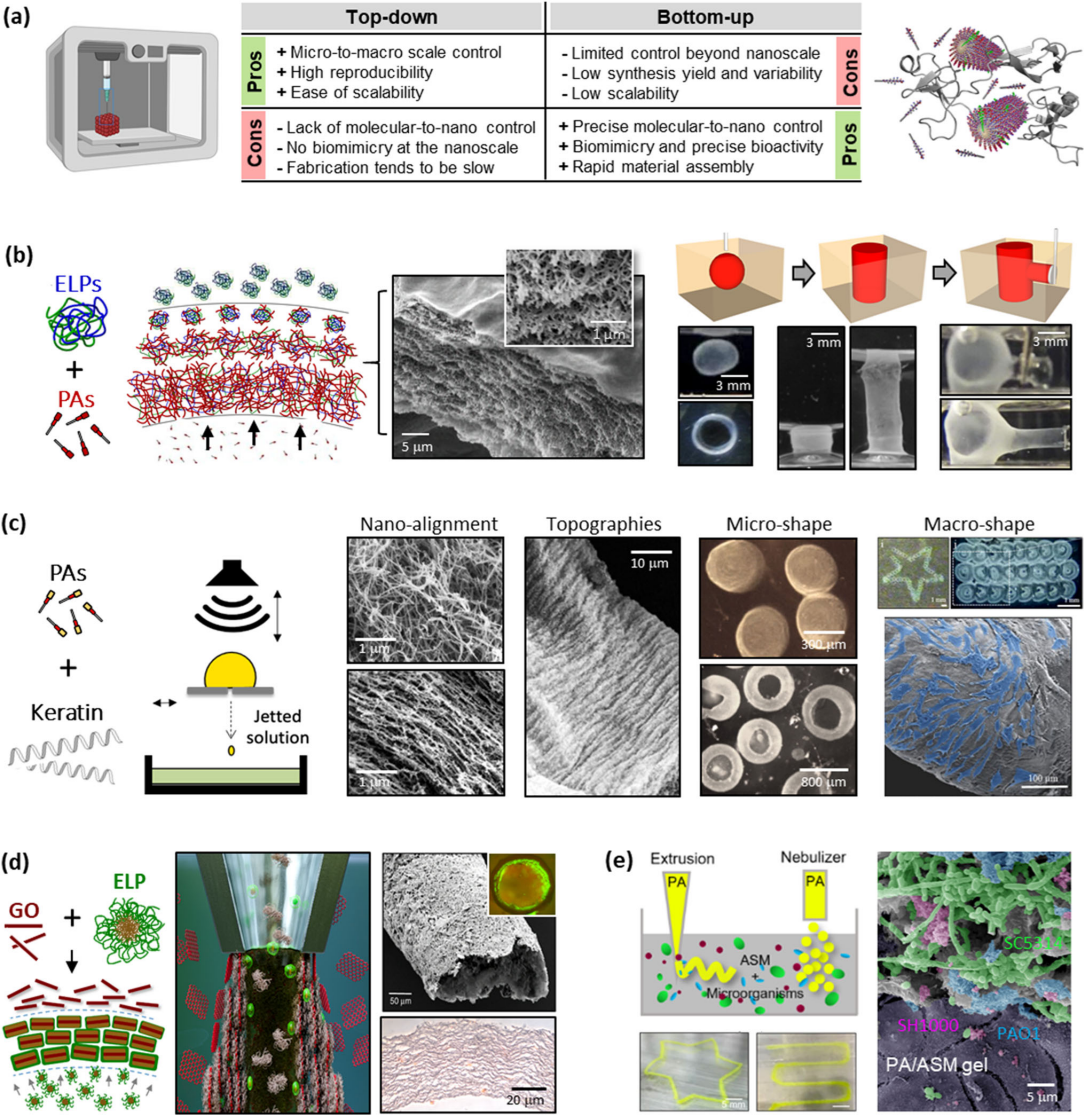
Supramolecular biofabrication. **a** Advantages and disadvantages of merging top-down (e.g., biofabrication) and bottom-up (e.g., supramolecular self-assembly) strategies. Reproduced from Ref. [106], Copyright 2010, with permission from Elsevier. **b** Through diffusion–reaction mechanisms, the supramolecular assembly of peptide amphiphiles (PAs) and elastin-like proteins (ELPs) gives rise to hierarchical-organised membranes and allows liquid-in-liquid fabrication of dynamic tubular structures. Reproduced from Ref. [99] (Copyright 2015, with permission from Macmillan Publishers Limited) and Ref. [107] (Copyright 2023, with permission from the authors, licensed under CC BY 4.0). **c** Drop-on-demand printing, with its involved hydrodynamic forces, is exploited to create PA-keratin composites with dictated fibre nano-alignment, surface topography, and macroscopic geometries. Reproduced from Ref. [102], Copyright 2018, with permission from WILEY-VCH Verlag GmbH & Co. KGaA, Weinheim. **d** Graphene oxide (GO) flakes co-assembled with ELPs are harnessed to grow and bioprint vascular-like perfusable tubes. Reproduced from Ref. [103] (Copyright 2020, with permission from the authors, licensed under CC BY 4.0) and Ref. [104] (Copyright 2021, with permission from the authors, licensed under CC BY 4.0). **e** Co-assembly of PAs and artificial sputum enables the creation of printable 3D models of bacterial infection. Reproduced from Ref. [105], Copyright 2023, with permission from the authors, licensed under CC BY 4.0

Prof. Alvaro Mata at the University of Nottingham is investigating supramolecular biofabrication methods that use biological organisation principles, such as self-assembly, compartmentalisation, and diffusion–reaction phenomena, as fabrication steps of manufacturing methods such as printing [90] and microfabrication [91]. This idea aims to synergise bottom-up and top-down methodologies to enhance the assembly of molecular and cellular components into functional structures by harnessing the advantages of one approach to overcome the disadvantages of the other (Fig. 5a) [90].

A first step towards this vision has been the use of self-assembling peptides as bioinks to facilitate the bioprinting of bioactive nanofibrous environments with micro- and macro-scale control [92, 93]. This approach can also enable control over nanofibre alignment [94, 95]. To enhance compositional diversity, peptide amphiphiles (PAs) have been co-assembled with ECM components into composite PA-ECM nanofibres capable of growing functional multicellular spheroids [96, 97]. This co-assembling approach can also be tuned to trigger compartmentalisation, diffusion–reaction phenomena, or protein disorder-to-order transitions, thus opening opportunities for controlling supramolecular assembly at higher size scales [89]. For instance, combining PAs with hyaluronan, sacs and membranes with hierarchical structure can be formed [98], while co-assembling PAs with elastin-like proteins (ELPs) generates multilayered membranes that can be manipulated into dynamic tubular networks (Fig. 5b) [99]. These supramolecular events can give rise to emerging properties such as the capacity to self-heal [99] or organise components such as graphene oxide (GO) flakes [100].

This multi-component self-assembling approach can be easily incorporated within liquid-in-liquid printing to provide higher levels of geometrical control and reproducibility [101]. For example, using drop-on-demand printing, co-assembly leads to PA-keratin nanofibres, while hydrodynamic forces taking place during the printing process were used to regulate nanofibre alignment, gel shape, gel surface topography, and macroscopic geometries (Fig. 5c) [102]. This approach can be used with other co-assembling systems, such as ELPs with GO, where liquid–liquid interfaces can be manipulated to simultaneously assemble and bioprint functional vascular-like structures (Fig. 5d) [103, 104]. Furthermore, these methodologies can be used with complex fluids such as artificial sputum media to bioprint in vitro models of infection (Fig. 5e) [105].

## North West England

### Williams Group, University of Liverpool

**Fig. 6:**
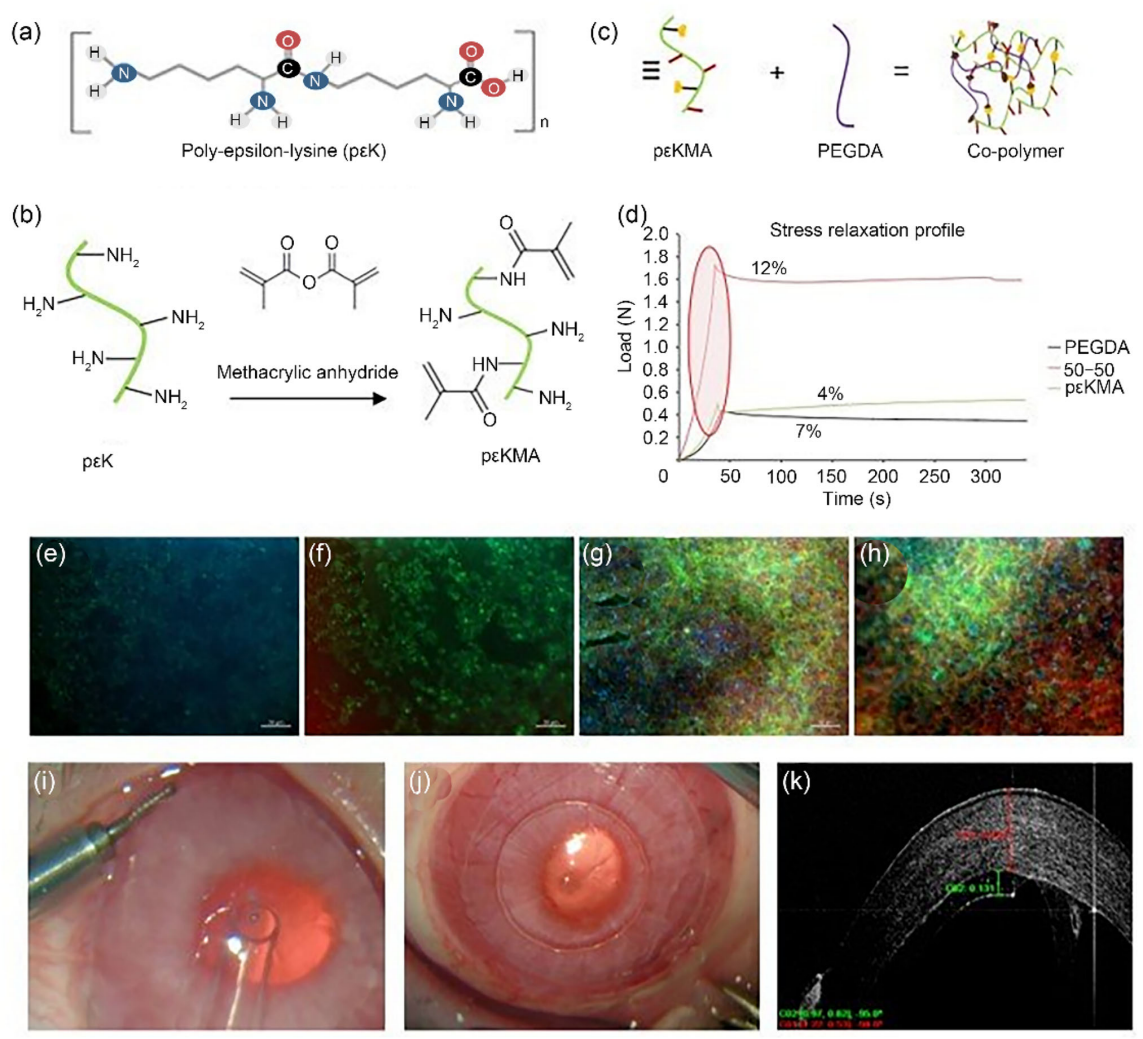
Fabrication of corneal hydrogels. **a** Poly-epsilon-lysine forms the basis of the hydrogels (p$\varepsilon$K). **b** p$\varepsilon$K is methacrylated by reacting *m* p$\varepsilon$K with methacrylic anhydride and triethylamine at pH 7 and 20 °C for 12 h to produce p$\varepsilon$KMA. Adapted from Ref. [108] (Copyright 2014, with permission from the authors, licensed under CC BY 4.0) and Ref. [109] (Copyright 2018, with permission from the authors, licensed under CC BY 3.0). **c** A co-polymer is formed from p$\varepsilon$K and poly(ethylene glycol) diacrylate (PEGDA). **d** The mechanical properties are improved for co-polymer hydrogels compared to p$\varepsilon$K and PEGDA alone. Cell attachment is affected by the formulation of the hydrogel as demonstrated using a human corneal endothelial cell line (HCEC12 cells). HCEC12 cells on **e** 100% p$\varepsilon$K hydrogel, **f** 90% p$\varepsilon$K/10% PEGDA, **g** 80% p$\varepsilon$K/20% PEGDA, and **h** 50% p$\varepsilon$K/50% PEGDA, after 7 d in culture (ZO-1 red, Phalloidin green, DAPI blue). The mechanical properties of the hydrogel are optimal for handling and delivery. **i** The hydrogel being delivered to the rabbit eye using an intraocular lens injector device and **j** after easy unfolding and centration in the anterior chamber. **k** The excellent positioning and attachment of the graft to the posterior rabbit cornea can be seen using optical coherence tomography (red indicating cornea and green graft). DAPI: 4',6-diamidino-2-phenylindole

Prof. Rachel Williams and co-workers at the University of Liverpool have developed a peptide hydrogel based on poly-*ε*-lysine (p*ε*K), which is composed of 25–30 amino acids with the peptide bond between the carboxylic acid on the central carbon and the $\varepsilon$-amine group on the end of the side chain (Fig. 6a). This produces a positively linear charged peptide with the $\alpha$-amine groups available for functionalisation and cross-linking to produce a gel. The Williams Group has developed a methodology to functionalise a percentage of the $\alpha$-amine groups with methacrylate groups (p$\varepsilon$KMA) to promote ultraviolet (UV) cross-linking (Fig. 6b). Using this technique, they have synthesised a family of hydrogels with varying mechanical and surgical handling properties by combining p$\varepsilon$KMA with poly(ethylene glycol) diacrylate (PEGDA) (Fig. 6c), demonstrating an increase in peak load for a 50:50 mix and an improved viscoelastic profile in comparison with p$\varepsilon$KMA or PEGDA alone (Fig. 6d).

The standard treatment for corneal endothelial failure is a cadaveric donor tissue graft, but there is a need to develop alternatives due to a global donor cornea shortage. To create a biosynthetic graft, a p$\varepsilon$KMA hydrogel was used as a carrier for a monolayer of in vitro expanded corneal endothelial cells (CECs). The ratio of p$\varepsilon$KMA to PEGDA influences the attachment and growth of CECs on the surface of the hydrogel; this is demonstrated in Figs. 6e–6h, which show human corneal endothelial cell line (HCEC12) cells on different formulations of the hydrogel. The 50:50 hydrogel enables the best cell attachment and production of a monolayer of cells with a cobblestone appearance as indicated by immunochemical analysis of ZO-1 expression at tight junctions (Fig. 6h).

Creating a hydrogel that has customisable mechanical properties allows for the material to be fine-tuned for specific medical uses. Current endothelial keratoplasty techniques such as Descemet’s membrane endothelial keratoplasty require considerable surgical skill when it comes to graft preparation, loading, and unfolding in the anterior chamber and attachment to the posterior stroma. In vivo, rabbit studies have demonstrated that the biosynthetic graft can be easily loaded and delivered using a clinical delivery device and, importantly, due to optimised material stiffness, instantly unfolds with minimal manipulation and securely attaches to the posterior stroma (Figs. 6i–6k).

Biosynthetic corneal grafts created using defined peptide hydrogels and expanded CECs offer a promising future alternative to cadaveric corneal grafts that could overcome the donor shortage. At least 30 grafts can be produced from just one donor using this method, a great improvement over the current 1-donor 1-recipient strategy.

### Hooper Group, University of Manchester

Neurovascular dysfunction underlies dementias such as Alzheimer’s disease and vascular dementia. The neurovascular unit consists of multiple cell types, including endothelial cells, pericytes, astrocytes and neurons embedded within the ECM of the brain. One of the key roles of pericytes is to support the blood–brain barrier function of the endothelial cells. To accurately recapitulate these complex cell–cell and cell–ECM interactions in vitro, 3D models require the use of hydrogels that mimic the physical and biochemical properties of the native ECM in which the different cell types can be co-cultured [110].

**Fig. 7:**
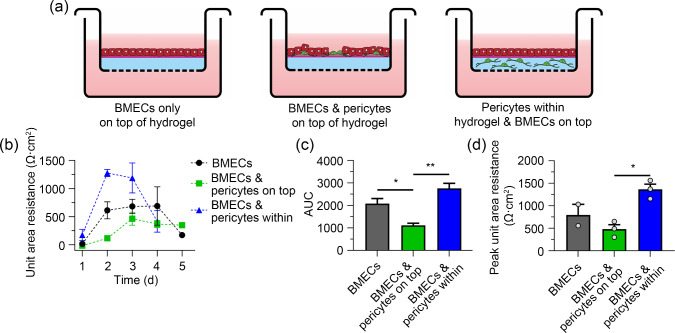
Pericytes embedded in hydrogel support the barrier function of brain microvascular endothelial cells (BMECs). OX1-19 induced pluripotent stem cells (iPSCs) were differentiated into BMECs and pericytes as described [112, 113]. PureCol hydrogel was layered on the surface of a Transwell insert with a coating of Matrigel on the upper surface. **a** BMECs were either cultured as a monoculture on top of the hydrogel, or mixed with pericytes on top of the hydrogel, or the pericytes were encapsulated within the hydrogel with the BMECs layered on top. Transendothelial electrical resistance (TEER) measurements were taken daily for five days and plotted as **b** unit area resistance (UAR) on respective days, **c** area under the curve (AUC) of **b**, and **d** peak UAR of **b**. TEER measurements were taken using an EVOM2 voltmeter and STX3 electrodes (World Precision Instruments, UK). Peak UAR is the highest recording of TEER of the whole measured time period. AUC and peak UAR were analysed using one-way analysis of variance (ANOVA) with Tukey’s multiple comparisons test. Data are shown as mean $\pm$ SEM. ^*^P $\le$ 0.05, ^**^P $\le$ 0.01. SEM: standard error of the mean

At the University of Manchester, Prof. Nigel Hooper employed collagen-based hydrogels that have a similar stiffness (approximately 1 kPa) to the brain ECM and promote cell–matrix interactions, to explore the ECM and spatial distribution requirements for the co-culture of brain microvascular endothelial cells (BMECs) and pericytes [111]. The spatial distribution of the cells of the neurovascular unit is also critical, as seen when BMECs are co-cultured with pericytes (Fig. 7). In the absence of pericytes, BMECs, when layered on top of a collagen hydrogel, formed tight junctions and an effective barrier as measured by transendothelial electrical resistance (TEER). When the pericytes were co-cultured with the BMECs and layered on top of the hydrogel, there was a decrease in TEER, whereas when the pericytes were incorporated into the hydrogel and then the BMECs layered on top, the TEER increased, indicating a better blood–brain barrier formation. These data highlight that (1) appropriate hydrogels which mimic the physical and biochemical properties of the brain ECM and (2) an appropriate spatial distribution of the neurovascular unit cells in the hydrogel are required to more accurately model neurovascular unit structure and function.

## London, England

### Gautrot Group, Queen Mary University of London

**Fig. 8:**
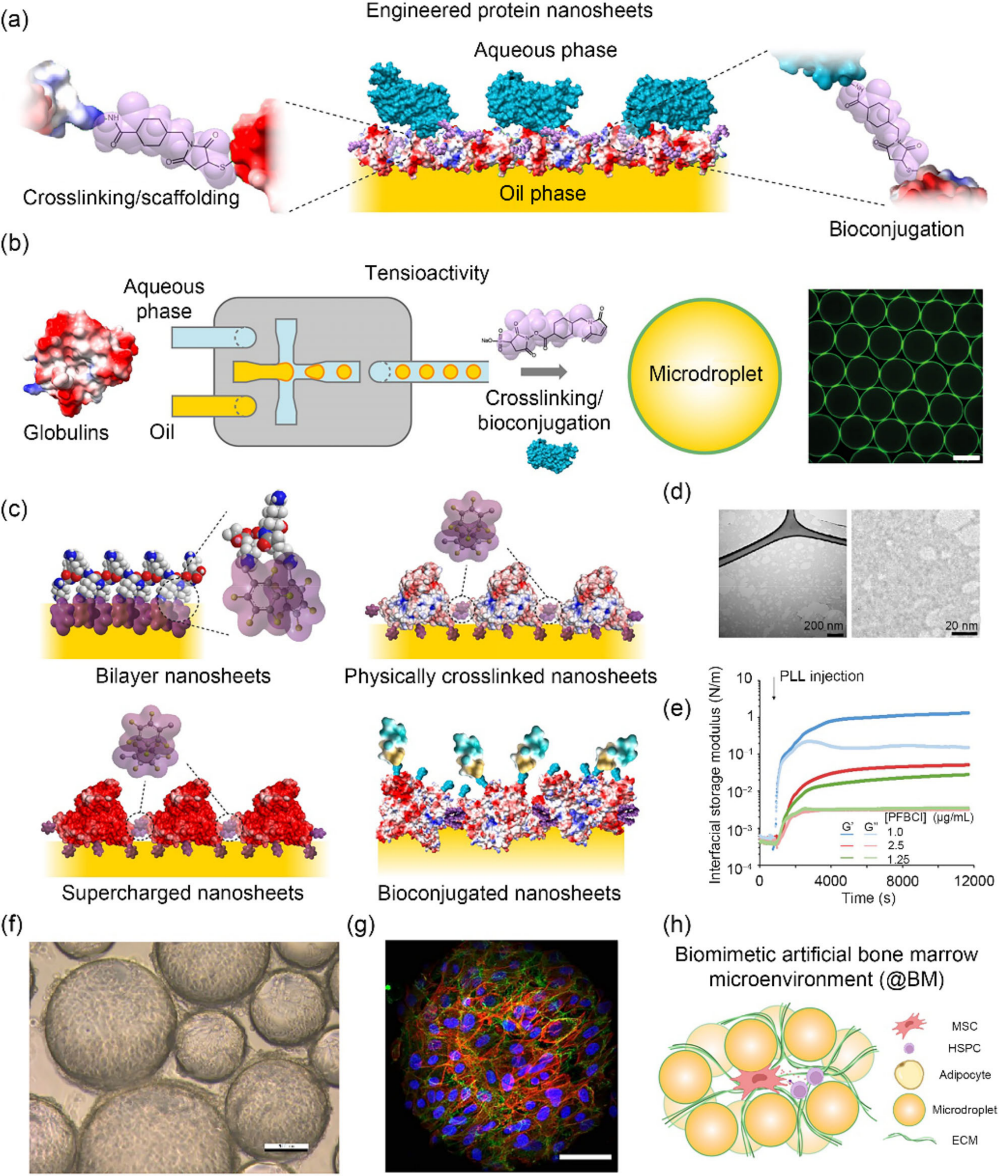
Biomanufacturing using microdroplet and protein nanosheet technologies. **a** Schematic representation of engineered protein nanosheets. Reproduced from Ref. [131], Copyright 2024, with permission from the authors, licensed under CC BY. **b** Protein nanosheet assembly can be orchestrated in microfluidic platforms to control the size of microdroplets. **c** Examples of protein nanosheet designs. Adapted from Ref. [124], Copyright 2023, with permission from the authors, licensed under CC BY 4.0. **d** Transmission electron microscopy images of protein nanosheets (albumin-based). **e** Changes in interfacial shear mechanics taking place upon assembly of poly(L-lysine) nanosheets at a liquid–liquid interface. Reproduced from Ref. [122], Copyright 2022, with permission from the authors, licensed under CC BY 4.0. **f** Brightfield microscopy image of HEK293 cells growing on a bioemulsion. **g** Colony of mesenchymal stem cells growing on a microdroplet (blue, nuclei; red, F-actin; green, vinculin). Reproduced from Ref. [123], Copyright 2023, with permission from the authors, licensed under CC BY 4.0. **h** Schematic representation of a microdroplet-based bone marrow microenvironment. Reproduced from Ref. [133], Copyright 2023, with permission from the authors, licensed under CC BY 4.0

Advances in stem cell technologies are revolutionising regenerative medicine and the pharma industry. However, an important hurdle to the broader translation of these technologies remains our limited ability to scale up and automate cell manufacturing and processing [114, 115]. Ninety-five percent of cell lines are anchorage-dependent and require solid and hydrogel microcarriers for expansion and differentiation into defined lineages [116]. These microcarriers require separation from the cell products and present potential sources of contamination that are unacceptable for translation. Although these issues can be managed at a lab scale, translation requires the scale up of cell production by more than 2–3 orders of magnitude [117, 118]. To bypass these hurdles, microdroplet technologies are attractive as they can be easily separated from cell masses by centrifugation or filtration. To enable cell adhesion to these liquid microcarriers (oil droplets), the controlled assembly of protein nanosheets was proposed by Prof. Julien Gautrot at Queen Mary University of London (Fig. 8) [119–121]. To sustain cell adhesion, resist cell-mediated contractile forces, and maintain cell phenotype, the proteins assembled at the corresponding liquid–liquid interfaces are required to display a combination of three key properties [122–124]. Firstly, they must be tensioactive in order to stabilise oil microdroplets. Secondly, they need to exhibit bioactive behaviour to enable the ligation of specific membrane receptors regulating adhesion (i.e., integrins). Thirdly, scaffolding must confer sufficiently strong mechanical properties to liquid–liquid interfaces to resist cell-mediated contractile forces. This led to the definition of bioemulsions: microdroplets stabilised by inherently bioactive amphiphiles.

The control of the interfacial shear mechanical properties (in the plane of the interface rather than in the normal direction), including the storage modulus, viscoelasticity and toughness of liquid–liquid interfaces, was found to be critical for sustaining cell adhesion and expansion on liquid substrates [120, 122, 125, 126]. This is in excellent agreement with the distribution of forces exerted by cells at lamellar protrusions or through focal adhesions, which are predominantly oriented in the plane of the cell membrane and substrate [127, 128]. In turn, engineered protein nanosheets were found to enable the long-term maintenance of mesenchymal stem cells (MSCs) upon culture on bioemulsions [129, 130]. Induced pluripotent stem cells adhering to bioemulsion microdroplets formed large colonies retaining pluripotency markers and the capacity to differentiate in defined lineages (i.e., cardiomyocytes) [131]. The secretory phenotype of cells cultured on bioemulsions, including cytokines, growth factors and exosomes, was also found to be stimulated [132, 133]. In turn, the unique microenvironment that MSCs remodel upon culture on microdroplets was harnessed to mimic the adipose-rich bone marrow niche and allow the maintenance of haematopoietic stem cells in a scalable form [133]. Overall, protein nanosheets and bioemulsions illustrate how the engineering of the nanoscale mechanics and biochemistry of biointerfaces can enable the maintenance of stem cell phenotype and offer unique design opportunities for novel biomanufacturing technologies.

### Li Group, The Francis Crick Institute

**Fig. 9:**
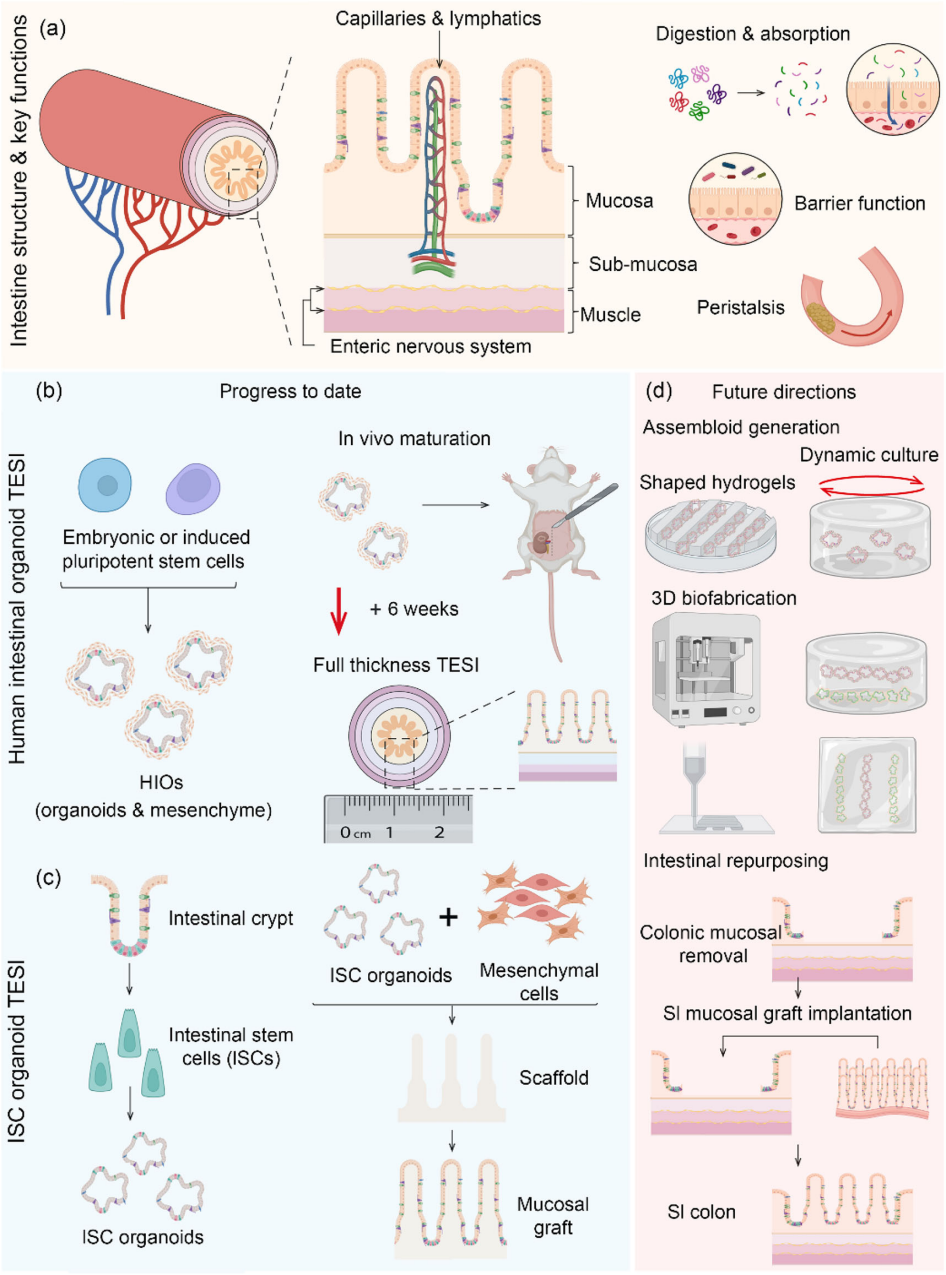
Intestinal tissue engineering—progress to date and future directions. **a** Schematic depicting intestinal structure and function, the predominant engineering strategies to date and proposed future directions. The intestine, a hollow tubular organ, has an inner mucosa, consisting of epithelium with supporting mesenchyme, a sub-mucosa containing vasculature and lymphatics, surrounded by circular and longitudinal muscle layers regulated by an enteric nervous system. It has various key functions including digestion and absorption, transit via peristalsis and the maintenance of a barrier against luminal micro-organisms. **b**, **c** Predominant intestinal engineering strategies to date include full-thickness tissue-engineered small intestine (TESI) from human intestinal organoids (HIOs) and mucosal grafts engineered from human intestinal stem cell (ISC) organoids. **d** Future directions include 3D biofabrication and culture techniques to increase engineered graft size and mucosal repurposing to generate a segment of small intestinalised colon. Figure created using BioRender.com

Intestinal tissue engineering (ITE) is a rapidly evolving field that combines stem cell and developmental biology, material science, and bioengineering. At the Crick Institute, Dr. Vivian Li is aiming to use ITE to address the unmet clinical need in the treatment of intestinal failure while also providing innovative in vitro modelling of gastro-intestinal (GI) tract development and disease. Tissue-engineered intestine (TEI) approaches seek to recapitulate both the architecture and function of the native intestine (Fig. 9a); currently, TEI models are most advanced for the small intestine (SI). Organoid technology, initially with stromal-free human intestinal stem cell (ISC)-derived intestinal organoids [134] and subsequently with pluripotent stem cell-derived human intestinal organoids (HIOs) [135], has accelerated progress in ITE over the past 10 years. ITE approaches using HIOs, comprised of cells from both endodermal and mesodermal lineages, are the most advanced at generating full-thickness, multilayered TEI. This approach relies upon in vivo maturation following heterotopic transplantation (Fig. 9b) [136]. Historically, HIO TEI required additional cell types, such as neural crest and endothelial cells, to generate enteric nervous system and vasculature [137–139]; however, recent evidence under peer review suggests that these additional components can be generated from HIO in vitro and matured in vivo to give rise to functional neural and vascular networks [140]. With microarchitecture closely mimicking the native intestine, HIO TEI is useful for modelling development and disease [139, 141]. However, given its relatively small size (a couple of centimetres), future therapeutic challenges lie in its potential for upscaling to generate TEI, which can be orthotopically transplanted. Achieving this will need to utilise 3D biofabrication and culture techniques in vitro to generate larger intestinal assembloid-like structures [142–144] prior to further maturation in vivo (Fig. 9d).

Alternative TEI approaches utilise ISC-derived organoids in combination with cell-free or mesenchymal cell-laden scaffolds (Fig. 9c) [145]. Dr. Li’s group has used such strategies to enable successful epithelial and mucosal reconstruction, generating functional grafts from patient-derived cells [146]. Despite this, engineering a full-thickness TEI for transplantation has not yet been accomplished. To be achieved, this would require the complexity of either additional primary cell types, including neural crest and smooth muscle precursors, or combination with HIOs. Organoid therapies have illustrated the feasibility of an alternative ISC organoid-based strategy, namely, the transformation of a segment of colonic epithelium to SI utilising the functional redundancy of the colon [147]. This can serve to expedite clinical translation and, in the future, be employed with in vitro SI mucosal sheet engineering for in vivo transplantation (Fig. 9d).

### Phillips Group, University College London

**Fig. 10:**
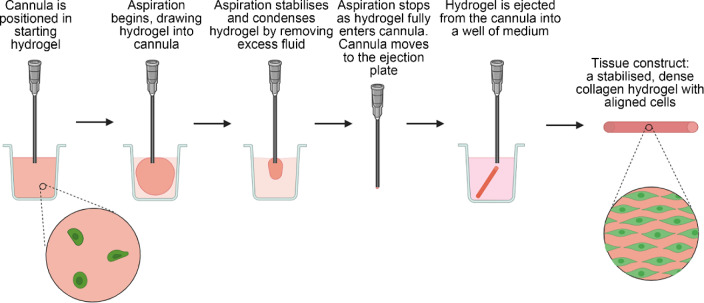
Flow diagram of the gel aspiration-ejection (GAE) method for biofabrication of engineered tissue constructs. Figure created using BioRender.com

The construction of living artificial tissues offers a promising option for regenerative medicine, where replacement tissues are required following injury or disease. An example of where an engineered tissue can address a severe unmet clinical need is in nerve repair, where current surgical approaches for repairing gaps rely on autografting, which has severe limitations, including tissue availability and donor site morbidity. Prof. James Phillips at University College London has developed engineered neural tissue (EngNT) to mimic key efficacious features of the autograft, using an aligned cellular collagen hydrogel with tissue-like density to provide support and guidance to regeneration of neurons [148, 149]. Scalable, cost-effective biomanufacturing methods of producing EngNT are essential for successful commercialisation and clinical translation [150]. A process has been developed that confers alignment on cellular hydrogels while simultaneously increasing their density through the removal of fluid; this involves aspiration into a cannula, with subsequent ejection of a fully-formed stable cylindrical tissue [151] (Fig. 10). The gel aspiration-ejection (GAE) approach offers an opportunity for automation through a programmable syringe pump, enabling high-quality, reproducible manufacturing [152]. The cannula and starting hydrogel size are customisable elements that permit the dimensions and mechanical properties of the resulting tissue construct to be tailored to a given application. In the case of EngNT, this method could be deployed in a cleanroom environment in accordance with Good Manufacturing Practice, as required for regulatory approval as an advanced therapy medicinal product (ATMP). Having an automated approach for EngNT manufacturing also provides the potential for future industrial-scale applications. Incorporating multiple syringe pump-controlled cannulas in parallel could generate large batches of constructs, and the deployment of identical equipment to manufacturing sites in different regions could reduce supply chain distances. Engineered tissues and ATMPs, in general, can be perceived as being highly complex with inevitably high manufacturing costs. Therefore, simple and rapid systems to produce living artificial tissues provide a valuable approach for low-cost biomanufacturing at scale, increasing the commercial feasibility and providing opportunities for clinical translation.

## East of England

### Biointerface Group, University of Cambridge

**Fig. 11:**
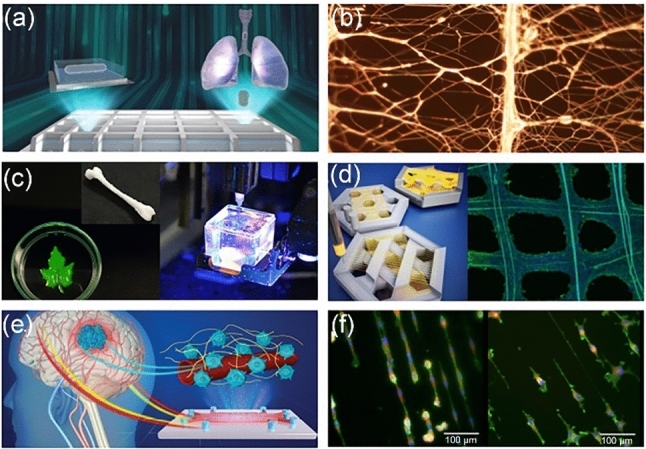
Multiscale biofabrication techniques. **a** Bio-assembling macro-scale, lumised airway tubes (reproduced from Ref. [158], Copyright 2021, with permission from the authors, licensed under CC BY). **b** Suspended piezoelectric nanofibre networks for acoustic sensing. **c** 3D printing of soft biomaterials. **d** 3D printing suspended fibres for cell culture (reproduced from Ref. [163], Copyright 2019, with permission from the American Chemical Society). **e** Microfluidic cell culture with microvessels (reproduced from Ref. [154], Copyright 2021, with permission from the authors, licensed under CC BY 3.0). **f** Cell ‘morphing’ on extracellular matrix analogues (reproduced from Ref. [164], Copyright 2014, with permission from the authors, licensed under CC BY)

At the University of Cambridge, Prof. Shery Huang’s ‘Biointerface group’ is developing 3D bioprinting and biofabrication techniques, focusing on sustainability and healthcare translatability [9, 19, 153]. The Biointerface Group takes a multiscale approach to fabricating microvessels [154], 3D printed biomimetics [155], and fibre-of-thing networks [9] (Fig. 11).

Bioprinting is an increasingly popular technology capable of 3D printing living tissues. It is a highly versatile tool that can be useful in modelling and investigating nearly any organ system. Major limitations that often hinder the deployment of this technology include the cost of entry and specialised engineering; most printers are costly and custom-made with specific applications in mind. To overcome these limitations and help democratise 3D bioprinting, Printer.HM, an open-source, hackable, and multi-functional soft material printer, was developed based on a robotic arm [156]. Another limitation of 3D printing is mobility. 3D bioprinters are traditionally large, static machines that are difficult to move and set up; this means that they cannot be shared between sites or used in temporary locations. For this reason, a transportable ‘BioArm’ has been developed. This ‘BioArm’ has been utilised to print tumouroids with cancer-associated fibroblasts in a core–shell configuration [157]. When fabricating soft hydrogels that are not self-supporting, a technique known as embedded 3D printing is used; this technique prints the bioink within a supportive bath. Choosing complementary bioink and supportive bath materials is essential in ensuring a viable print; however, there is no reliable, well-established technique to guide bath selection. The Biointerface Group recently developed a set of guidelines for selecting the bath composition based on the bioink used [31]. This library can improve the efficacy of prints and speed up the design of embedded bioprinting experiments while allowing access to a broader range of hydrogel materials.

In order to fabricate scaffold-free airways, a bioassembly based technique known as multi-organoid patterning and fusion (MOrPF) has been developed [158]. This technique fuses multi-organoid aggregates to allow for the fabrication of epithelial tubes in customisable geometries that enable fluid flow and can serve as vessel building blocks for multi-organ systems. Additionally, the Biointerface Group has fabricated microfluidic devices that allow cell culturing of distinct cell populations in synthetic microvessels [154].

Fabricating a conductive fibre-of-things network is often difficult due to the fragility of the fibres and a lack of connections between fibres. Using traditional fabrication techniques, it is difficult to manipulate individual fibres without breaking them when connecting them into a circuit, which often results in a large increase in resistance, rendering them unusable. Inflight printing allows for the formation of in situ bonds between thin conducting fibre arrays [159–161]. These emerging fibre fabrication techniques can pave the way towards a sustainable wearable electronics revolution [9, 162].

Presently, the Biointerface Group is leveraging these biofabrication and 3D bioprinting techniques as sustainable technologies for healthcare, electronics, and engineering biology. Current topics of investigation include biodegradable electronics that can be deployed in situ without impacting the native environment, improved in vitro culture platforms for modelling the brain, lungs, and heart, and using biohybrid systems as biological sensors.

## South East England

### Stevens Group, University of Oxford

**Fig. 12:**
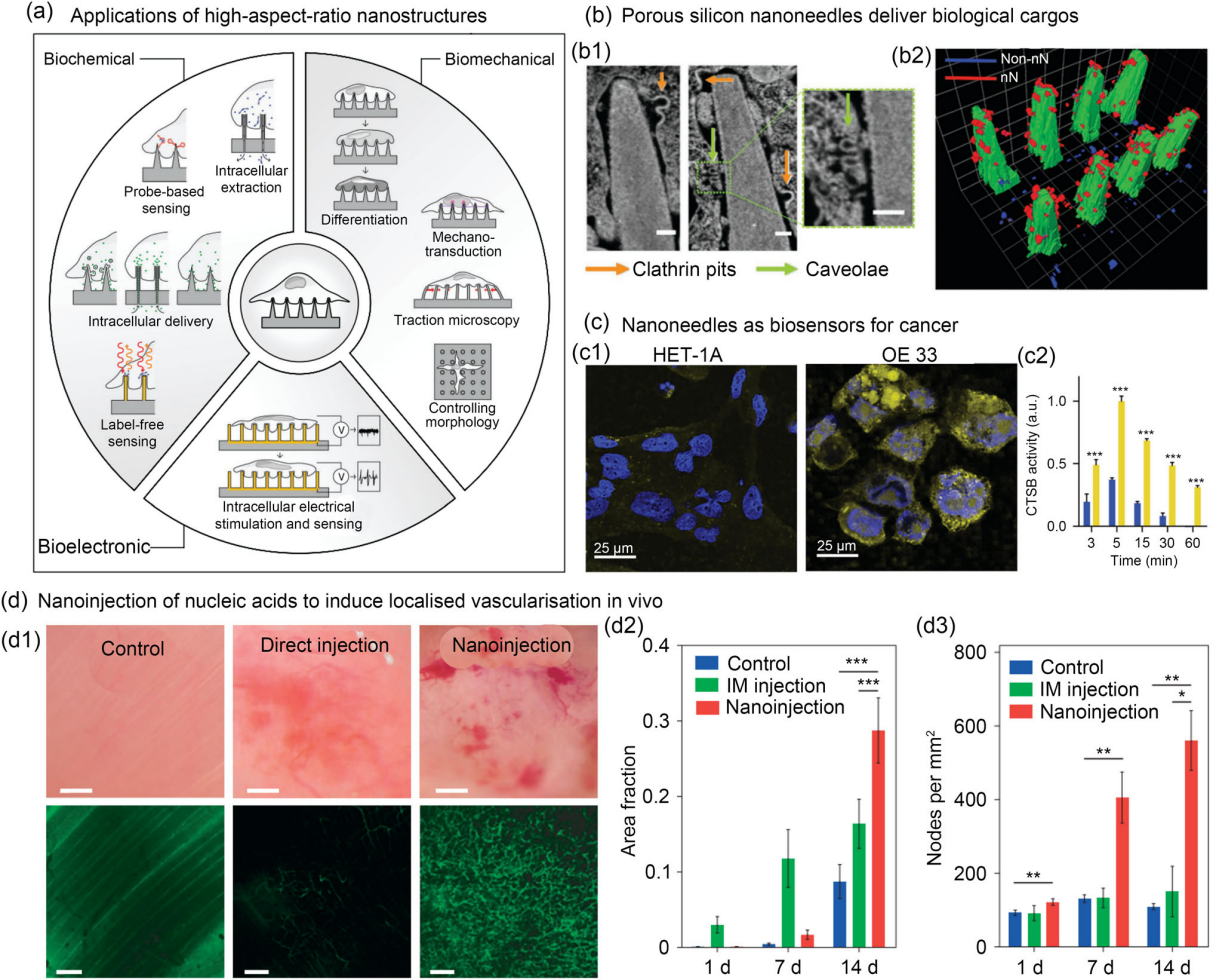
Engineered nanotopographies as minimally invasive nanotools for cargo delivery, biosensing, and in vivo cell reprogramming. **a** Schematic representation of the diverse applications of high-aspect-ratio nanostructured surfaces which leverage intimate contact between the substrate material and cell membrane (reproduced from Ref. [165], Copyright 2020, with permission from WILEY–VCH Verlag GmbH & Co. KGaA, Weinheim). **b** Nanoneedles have been employed to deliver specific cargo which elucidated uptake mechanisms: **b1** focused-ion-beam scanning electron microscope (SEM) imaging demonstrated cell membrane interaction with nanostructures with the formation of two classes of endocytic vesicles clathrin pits (orange arrows) and caveolae (green arrows) (scale bars: 100 nm); **b2** the organisation of vesicle structures on nanoneedles (red) and non-nanoneedle locations (blue) was achieved using 3D reconstruction. Reproduced from Ref. [166], Copyright 2019, with permission from the authors, licensed under CC BY. **c** Nanoneedles have been used to detect cancerous cells (OE 33) based on cytosolic levels of cathepsin B (CTSB): **c1** representative images of a single *z*-plane through the cytosol of CTSB − ve (HET-1A) and + ve (OE 33) cells where yellow fluorescence arises from CTSB mediated cleavage of a fluorescent probe on the nanopatterned substrate and blue is the nuclei (scale bars: 25 µm); **c2** quantification of the area-normalised cytosolic fluorescence signal for OE 33 (yellow) and HET-1A (blue) when interfaced with nanoneedle sensors for various times (*x*-axis). Reproduced from Ref. [171], Copyright 2015, with permission from the authors, licensed under CC BY. **d** Nanoneedles have enabled the in vivo delivery of a growth-factor-encoding plasmid. **d1** Bright-field (top) and confocal (bottom) microscopy showing vasculature within muscles of untreated (control) and human vascular endothelial growth factor (hVEGF)-165 treated (direct-injection and nanoinjection). Fluorescent signal is from systemically injected fluorescein isothiocyanate (FITC)-dextran (scale bars: 100 µm for bright-field and 50 µm for confocal). Vessel quantification is demonstrated by **d2** the fraction of fluorescent signal and **d3** the number of nodes in the vasculature per mm^2^. Reproduced from Ref. [167], Copyright 2015, with permission from Macmillan Publishers Limited

Cell–material interactions are a fundamental consideration in biofabrication and biomanufacturing. While biointerfacing covers a range of length scales, high-aspect-ratio nanostructures represent a diverse and exciting field of research targeting cellular nanoinjection, biophysical stimulation, and biosensing (Fig. 12). Collectively, these efforts aim to leverage intimate interactions between the cell membrane and a patterned substrate to elicit numerous biological responses [165]. Engineered nanotopographies have been employed as minimally invasive nanotools for high-efficiency cargo delivery, such as nucleic acids. Prof. Molly Stevens has previously shown that porous silicon nanoneedle arrays enhance the internalisation of pathway specific payloads and nucleic acids into human stem cells via stimulation of independent endocytic pathways [166]. Similar arrays have enabled localised in vivo cell reprogramming, facilitating significantly higher levels of neovascularisation in muscle tissue compared to traditional direct injection. Importantly, this ‘nanoinjection’ induced enhanced expression of human VEGF165 for up to seven days with superior vessel formation, interconnection, and perfusion (sixfold increase) without inducing any local acute inflammation or tissue damage, demonstrating the capacity of nanoneedles to efficiently mediate in situ therapeutic delivery [167]. Recently, nanoneedle gene-delivery has been further enhanced by combination with self-assembled polyplex-polysaccharide nanofilms. Specifically, a nanometre-scale multilayer coating provided fine control over cargo loading and release kinetics, which correlated with transfection efficiency. The inclusion of polysaccharides increased transfection when compared to polyplexes alone and this synergistic effect resulted in cargo amounts a magnitude lower than those needed in standard transfection methods [168]. Such approaches are attractive clinically where spatially localised transfection with confined depth penetration is necessary, such as targeting tissue regeneration through activation of progenitor cells residing within defined tissue niches [168]. High-aspect-ratio nanostructured surfaces have also been employed to study and direct cell phenotype through biophysical interactions. Nanoneedles have been used to directly target intracellular elements responsible for mechanotransduction and have been shown to elicit simultaneous but distinct responses from the cell membrane, cytoskeleton, and nucleus of human cells [169]. The tunability of high-aspect-ratio nanostructures (tip diameter, pitch, tip length, density) facilitates precise investigation into the interplay between mechanical microenvironmental parameters and cellular behaviours in complex cell–material interactions, such as cell polarisation, morphological heterogeneity, nuclear morphology, and gene expression [170]. Moreover, by providing minimally invasive access to the intracellular environment, nanoneedles are positioned as potential diagnostic devices. Nanoneedle biosensors have been shown to be effective in mapping cancer cells, approaching single-cell resolution, within cell cultures and biopsied tissues. In this context, the nanostructured sensor was able to interface with the intracellular environment and sense a cathepsin B (CTSB, a known solid-tumour biomarker) activity via fluorescently labelled CTSB cleavable peptide covalently conjugated to the sensor’s surface [171]. Taken together, engineered nanotopographies represent a potent method to control cell behaviour, enabling direct interfacing with the cellular machinery. They have been demonstrated as effective methods for gene delivery, biophysical stimulation, and biosensing, highlighting the exciting translational potential of such nanotools.

The advancement of bioprinting technologies has made it a core technology in the field of biofabrication and biomanufacturing. The emergence of novel printing methods must also be matched by the generation of new printable materials, including bioinks. Bioinks are a class of materials containing biologics (e.g., cells, biomolecule, nucleic acid) that can be dispensed through a printhead without compromising the viability or functionality of the encapsulated biologic [172]. A persistent challenge in the development of functional bioinks is balancing their rheological and biological properties. Previously, the competing requirements of printability and biological needs have created a relatively narrow ‘biofabrication window’, whereby only a limited number of bioinks enabled the formation of high-fidelity structures and maximal biocompatibility [173]. However, in recent years, Prof. Stevens and others have explored the development of new classes of bioinks which extend the biofabrication window by providing the necessary physiochemical properties required for 3D deposition without compromising the functionality of the encapsulated biologics [174, 175]. Specifically, Prof. Stevens’ group has developed complementary network bioinks with gelation mechanisms, which enable control throughout the fabrication process. The combination of a thermo-responsive gel network with a photo cross-linkable secondary network underpinned the development of 20 different formulations using 12 different polymers, enabling 3D fabrication at lower bulk gel concentrations previously considered “unprintable” [174]. Alternatively, thermo-sensitive hydrogels can be employed as sacrificial materials for the generation of perfusable channels [32, 176]. Expanding on this concept, Prof. Stevens has generated a void-free 3D bioprinting method, which enables the generation of perfusable, endothelialised channels within a single-step printing process [177]. Mass transport limitations can significantly hinder the scalability of biofabricated tissues/grafts. The inclusion of lumens, channels, and porosity within constructs has gone some of the way to improving the diffusion of culture medium into the centre of constructs [178, 179]. However, creating micron-scale porosity within the bioinks could help to further enhance the exchange of nutrients and soluble biochemical factors to cells. Efforts to create porosity within printed bioinks have utilised gelatin microgels as a method of creating tuneable pores in cell-laden bioinks, resulting in pore sizes at the cell-scale (10 μm) and the generation of 20%–70% porous bioinks which were compatible with extrusion and light-based bioprinting technologies [180]. Bioinks with the ability to deform over time have led to the invention of ‘4D bioprinting’, where materials change shape triggered by a stimulus or through the action of cells within the construct post-fabrication [181–183]. A magnetic field can be used to trigger shape changes, which has enabled the creation of complex 3D structures, like the branching networks of vascular systems, from 2D bioprinted constructs [36]. Moreover, these magnetic bioinks can be used as biohybrid soft-actuators capable of locomotion driven by living cells [36].

### Cui and Ye Group, University of Oxford

Research of Prof. Zhanfeng Cui, Dr Hua Ye, and co-workers at the University of Oxford focuses on harnessing tissue engineering and stem cell biology technologies for regenerative medicine therapies targeting cancer [184] and neural degeneration [185, 186]. While stem cell therapies are becoming increasingly popular, they are prohibitively expensive. In order to lower costs and increase the accessibility to these platforms, membrane engineering can be harnessed in order to adjust the surface area and transport properties. This has been demonstrated in expanding CAR-T cells as well as producing extracellular vesicles to be used as delivery vectors [187]. With stem cell therapies, the delivery vectors are important for delivery, survival, and proliferation. Ca-alginate based biomaterials are popularly used as microcarriers for delivery; however, they require complicated functionalization. The research team has shown that using Fe-alginate macro- and micro-beads supports mesenchymal stem cell (MSC) proliferation without the need for added functionalization [188]. The team has also developed an injectable hyaluronic acid hydrogel for neural regeneration [189]. This hydrogel uses hyaluronic acid as the central nervous system extracellular matrix and leverages dopamine to support dopaminergic neurons. Additionally, this hydrogel supports the culture of embedded human MSCs (hMSCs), serving as a central nervous system specific platform for stem cell transplantation that encourages repair and regeneration. Cell-laden granular scaffolds were used to improve cell viability and neurite extending compared to using bulk-hydrogels [185, 186]. This approach better recapitulates the neural microenvironment and improves long-term cell survival, differentiation, and maturation of neural cells which is essential for studying the forming of a complex neurite network. In vivo work was carried out to show that hyaluronic acid granular hydrogels effectively promote the morphological and functional recovery of the injured sciatic nerve [190]. Beyond hydrogel composition, the team has further performed extensive work on developing bioreactor systems for large-scale culture of therapeutic cells and tissues [191, 192]. Experimental and computational work was carried out to optimize bioreactors for a wide range of culture platforms including both adherent cell culture and suspension culture [193–195]. They have also shown that biomimetic soft membranes can be used to guide tissue regeneration in 3D space through control of pore geometry [196]. Furthermore, using aligned fibres as the culture substrate can encourage the directional growth of neurons [197]. These approaches improve the validity of in vitro culture platforms for studying neural networks and increase the clinical translatability of stem cell therapies.

### Dawson Group, University of Southampton

Nanoclays, particularly synthetic smectites such as Laponite, are emerging as materials of great promise for regenerative medicine due to their unique physical and chemical attributes. Nanoclays are characterised by single crystal layer platelets, less than 1 Å (1 Å=1×10^–10^ m) in height and about 25–30 nm in diameter. These exhibit a negatively charged face and a variably charged edge, which facilitates their self-assembly into a complex fractal gel structure upon suspension in water. Further, in the presence of ions and proteins, nanoclays assemble to form protein-rich gels able to support cell growth and differentiation, making these colloids a versatile medium for biomedical applications [198].

**Fig. 13:**
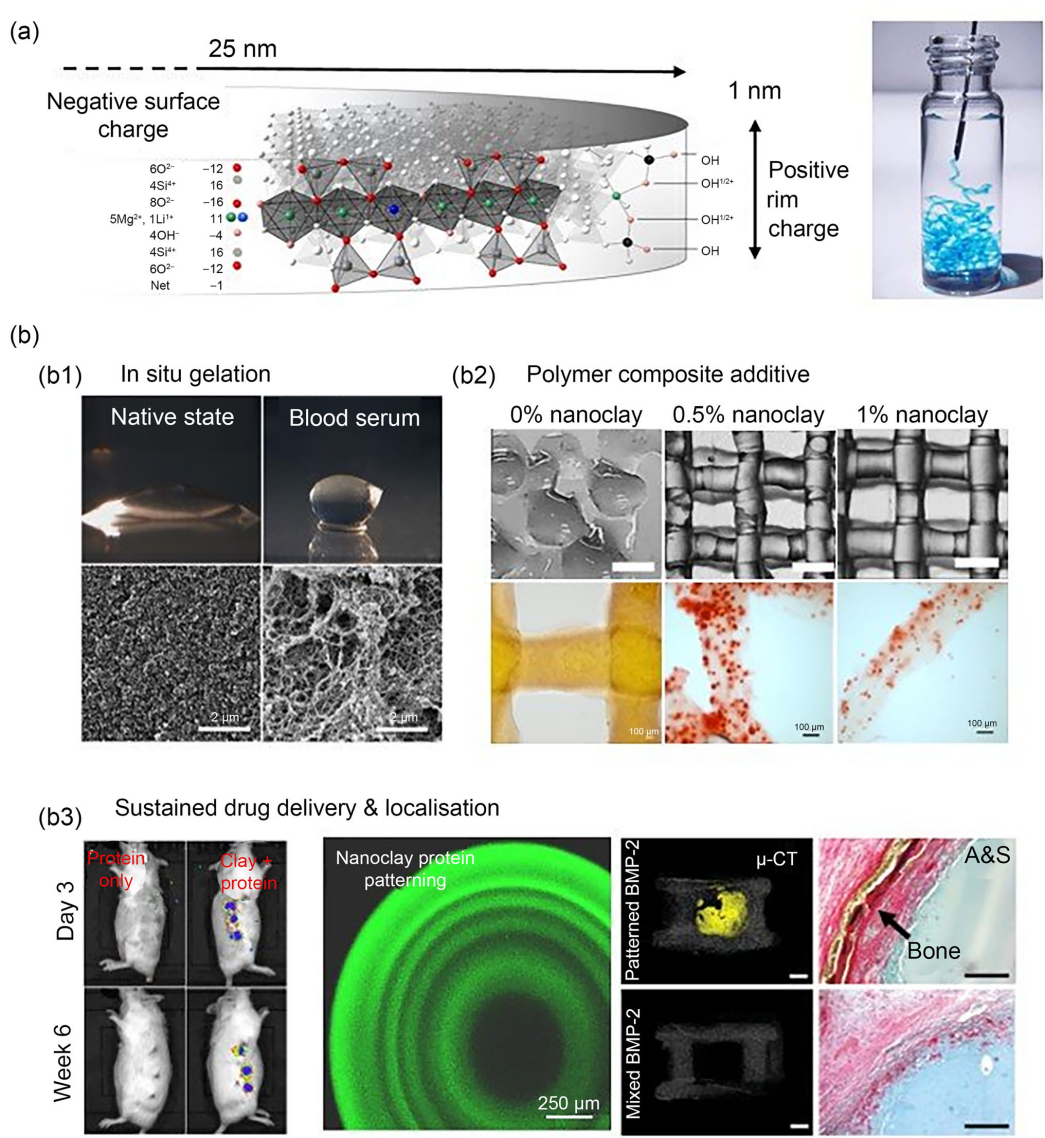
Harnessing nanoclay chemistry for regenerative medicine. **a** The synthetic clay Laponite consists of 25-nm diameter, 1-nm thick disks that possess a permanent negative surface charge and a pH dependent rim charge (positive below pH 9) (reproduced from Ref. [214], Copyright 2013, with permission from WILEY–VCH Verlag GmbH & Co. KGaA, Weinheim). **b** These properties generate a surprisingly rich array of possibilities for regenerative medicine. **b1** Aqueous solutions of nanoclay self-assemble into stiff gels upon contact with blood to form bioactive environments able to attract the invasion of stem cells ​(reproduced from Ref. [215], Copyright 2018, with permission from WILEY–VCH Verlag GmbH & Co. KGaA, Weinheim)​. **b2** The addition of nanoclay within hydrogel composites offers improved rheological properties as well as cell viability (reproduced from Ref. [201], Copyright 2019, with permission from IOP Publishing Ltd).​ **b3** Due to their affinity for proteins such as growth factors, nanoclay gels can sustain localised concentrations to promote new tissue formation in the body at reduced effective doses, and controlling diffusion reaction processes allows for high-resolution control over stable protein concentration gradients for direct tissue regeneration​ (the left part was reproduced from Ref. [202], Copyright 2020, with permission from the authors, licensed under CC BY 4.0; the right part was reproduced from Ref. [209], Copyright 2023, with permission from the authors, licensed under CC BY)

A key feature of nanoclay is its ability to immobilise biomolecules on its surface through physical adsorption while maintaining activity. At the University of Southampton, this feature has been applied by Prof. Jonathan Dawson’s group in vivo as a strategy to achieve localised, enhanced growth factor activity to promote bone formation [199] and vascularisation [200]. It is this ability, in particular, to functionalise nanoclays without harsh reactants or complex low yield conjugation chemistry that has opened up a surprisingly rich array of opportunities for regenerative medicine (Fig. 13).

Nanoclays have long been used as physical cross-linkers of polymers, to impart improved mechanical function in nanocomposites. Recently, nanoclays have been applied as rheological modifiers to fabricate various hydrogel polymer composites conducive to cell encapsulation and 3D extrusion printing [201]. Integrating nanoclays into bioinks has resulted in new compositions characterised by high print fidelity, enhanced cell viability, and improved tissue generation [201, 202].

Current research themes utilising nanoclay in biomedical contexts are diverse. Recently, Laponite coatings have been effective in delivering BMP-2 over eight weeks, contrasting with the short-lived burst release from collagen sponges [203]. Investigations into the interactions between Laponite and various cell types, including hMSCs, have demonstrated its direct bioactive effects in steering osteogenic and chondrogenic pathways [204, 205], as well as influencing immune cells like macrophages and dendritic cells, thereby modulating immune functions [206–208]. The advancement in protein patterning with microscopic precision within Laponite hydrogels marks a significant development. This allows for more localised protein delivery in regenerative scaffolds, moving from homogeneous biochemical signals to biomimetic gradients, thereby enhancing treatment efficacy [209].

Looking ahead, the unique adsorptive qualities of nanoclays are expected to gain wider application in biomanufacturing. These unique adsorptive qualities make nanoclays a powerful tool for protein immobilisation, maximising the efficacy of loaded agents while minimising dose with precision localisation. This, in addition to the expansion of the types of molecules being delivered, including enzymes [210], RNAs [211], and Au NPs [212] is an exciting prospect for new gene therapies, organoid production [213], and small molecule modification platforms.

## South West England

### Armstrong Group, University of Bristol

**Fig. 14:**
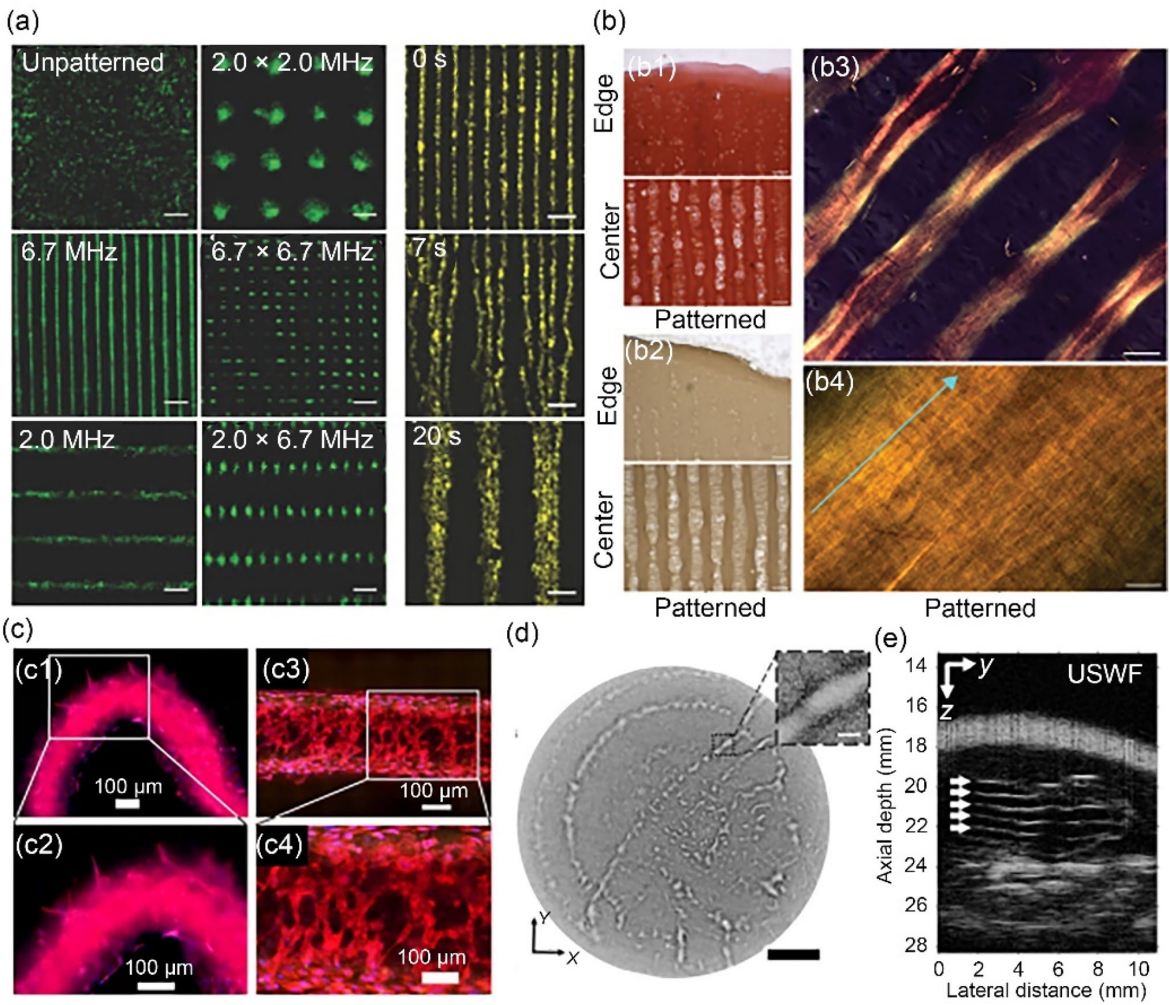
Acoustic cell patterning techniques and applications. **a** Fluorescence microscopy of acoustically patterned myoblasts, demonstrating the flexibility and rapid dynamic control of ultrasound patterning. Scale bars: 200 µm. Reproduced from Ref. [35], Copyright 2019, with permission from the authors, licensed under CC BY. **b** Histology showing the deposition of aligned collagen fibres by acoustically-patterned primary bovine articular chondrocytes in agarose: **b1** staining for sulfated glycosaminoglycan using safranin O, **b2** immunostaining for type II collagen, **b3**, **b4** polarized light microscopy following picrosirius red staining. Scale bars: 100 µm for **b1**, **b2**, 50 µm for **b3**, and 5 µm for **b4**. Reproduced from Ref. [220], Copyright 2022, with permission from the authors, licensed under CC BY. **c** Distinct regions of angiogenic sprouting (c1, c2) and cellular network formation (c3, c4) in differentially acoustically patterned regions of RFP-HUVEC-laden bioprinted constructs. Scale bars: 100 µm. Reproduced from Ref. [221], Copyright 2023, with permission from IOP Publishing Ltd. **d** Acoustic holographic patterning of HCT-116 cells within a collagen hydrogel. Scale bar: 5 mm; inset scale bar: 500 µm. Reproduced from Ref. [223], Copyright 2019, with permission from the authors, licensed under CC BY. **e** In vivo acoustic patterning of collagen-suspended GFP-HUVECs (reproduced from Ref. [226], Copyright 2023, with permission from the authors, licensed under CC BY 4.0). HUVEC: human umbilical vein endothelial cell; GFP: green fluorescent protein; RFP: red flourescent protein

At the University of Bristol, Dr. James Armstrong and co-workers are developing strategies to remotely assemble bio-functional components for tissue engineering and regenerative medicine [216, 217]. Of particular interest is the contactless manipulation of living cells using ultrasound standing waves [218] (Fig. 14a). Typically formed using pairs of opposing acoustic signals, standing waves offer the benefit of static pressure nodes and antinodes. Cells placed into an ultrasound standing wave experience an acoustic radiation force that, under certain conditions, can move those cells into the pressure nodes of the field [219]. A single ultrasound standing wave produces parallel lines of cells, which can be immobilised within hydrogel-based biomaterials. This general approach was used for the engineering of skeletal muscle [35] and deep-zone cartilage [220]. In both cases, the anisotropic cellular organisation led to enhanced tissue structure and function, namely the development of skeletal muscle with oriented myotubes and anisotropic tensile mechanics and the formation of hyaline cartilage with aligned collagen fibres (Fig. 14b). A focus of the Armstrong Group is to increase the accessibility and uptake of ultrasound-based cell manipulation in the biomedical sciences, which has so far entailed a University of Bristol spin-out (Impulsonics) and a practical summer school on “patterning cells with sound”.

This field of acoustic bio-assembly is now embracing new technological advances that are pushing the boundaries of ultrasound-based cell manipulation. There have been interesting developments that have expanded the capabilities of ultrasound technologies to generate more customised cell arrays. For instance, ultrasound standing waves can be combined with extrusion bioprinting to produce cell-patterned constructs within 3D printed geometries [221] (Fig. 14c). An alternative approach is to project ultrasound waves through topographically defined materials, which leads to phase offset and user-customised pressure fields [222]. These “acoustic holograms” have been used to generate complex cell assemblies in 2D [223] and 3D [224] space (Fig. 14d). Acoustic holography has also been used to pattern cells expressing intercellular gas vesicles, which inverts and magnifies the acoustic contrast of cells [225]. Finally, the field is also seeing a push to translate ultrasound technologies towards cell manipulation in vivo [226] (Fig. 14e). This concept takes advantage of the deep tissue penetration of ultrasound and, in the future, may be combined with other recent developments in ultrasound-triggered hydrogelation [227] and acoustic volumetric bioprinting [228] for minimally invasive clinical therapy.

## Scotland

### Shu Group, University of Strathclyde

**Fig. 15:**
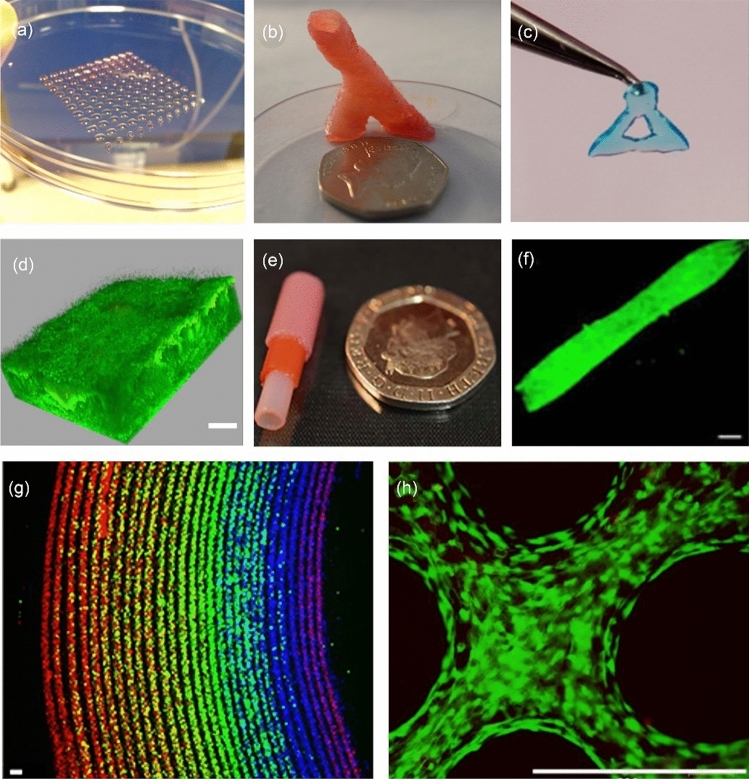
Biofabrication research in Shu’s lab: **a** 3D-bioprinted droplet array of human embryo stem cells (reproduced from Ref. [229], Copyright 2013, with permission from IOP Publishing Ltd); **b** 3D-printed robust, freestanding alginate hydrogel blood vessel-like structure using secondary (Ca^2+^) and tertiary (Ba^2+^) crosslinking steps (scale bar: 20 p coin) (reproduced from Ref. [231], Copyright 2015, with permission from the authors, licensed under CC BY 3.0); **c** 3D-printed polypeptide-DNA hydrogel with blue dye added for visualisation that is robust enough to be picked up by tweezers (reproduced from Ref. [232], Copyright 2015, with permission from Wiley–VCH Verlag GmbH & Co. KGaA, Weinheim); **d** 3D-reconstructed confocal laser scanning microscope *Z*-stack of *P. aeruginosa* biofilm in a 4-mm thick hydrogel construct following 14 days of maturation (scale bar: 100 µm) (reproduced from Ref. [233], Copyright 2019, with permission from the authors, licensed under CC BY 3.0); **e** multilayered alginate hydrogel tubular structure produced via a micro-dip coating method (scale bar: 20 p coin) (reproduced from Ref. [234], Copyright 2017, with permission from the authors, licensed under CC BY); **f** fluorescence image of a collagen-organoid tube for transplantable bile duct applications (scale bar: 100 µm) (reproduced from Ref. [236], Copyright 2017, with permission from Nature America, Inc., part of Springer Nature); **g** confocal image of multilayer prelabelled red, green, and blue high density human embryonic kidney (HEK) cells in a 1% (0.01 g/mL) alginate tube positioned via the rotational internal flow engineering (RIFLE) method (scale bar: 100 µm) (reproduced from Ref. [237], Copyright 2023, with permission from the authors, licensed under CC BY 4.0); **h** live/dead fluorescent imaging of murine adipose derived stem cells within a micromachined, electrospun polylactic-co-glycolic acid (PLGA) fibre mesh (scale bar: 500 µm) (reproduced from Ref. [238], Copyright 2023, with permission from the authors, licensed under CC BY 4.0)

Over the past decade, the field of tissue engineering and regenerative medicine has witnessed major developments owing to the remarkable advancements in biofabrication techniques, particularly in 3D bioprinting. Contributing to this revolutionary technology, Prof. Shu’s lab at the University of Strathclyde was the first to successfully demonstrate the bioprinting of human pluripotent stem cells, both embryonic and induced (Fig. 15a) that remained viable and pluripotent post-printing [8, 229, 230]. This break-through opened up several other research avenues, including the 3D-bioprinting of human tumour-derived cells that showed viability for up to 11 days when embedded within a robust and stable alginate hydrogel fabricated via a secondary (Ca^2+^) and tertiary (Ba^2+^) ionic-crosslinking strategy (Fig. 15b) [231]. Moreover, in order to produce hydrogels that could support extended cell viability, an innovative supramolecular polypeptide-DNA bioink was developed for 3D-bioprinting (Fig. 15c), with the resulting hydrogel demonstrating self-healing properties, shape retainability, and biodegradability [232]. Moving on from mammalian cells, recent work in Shu’s lab has also explored 3D-bioprinting of hydrogel-based bacterial biofilms for in vitro antimicrobial drug testing (Fig. 15d) [233].

While Shu’s research has mostly focussed on 3D bioprinting, other biofabrication techniques have also been investigated, specifically methods that do not involve complex machinery. A micro dip-coating method demonstrated for the first time the fabrication of multilayered, thin-walled (100–200 µm) cell-laden, alginate hydrogel tubular structures (Fig. 15e) [234, 235]. Further improvement on this method led to the production of collagen hydrogel tubular scaffolds comprising cholangiocyte organoids as potential transplantable bile ducts (Fig. 15f) [236]. Inspired by the multilayering of human tissue, as typically observed in histological samples, an innovative, low-cost, and tuneable rotational internal flow engineering (RIFLE) technique was recently developed to fabricate multilayer tissue-like constructs using cell-laden hydrogels (Fig. 15g) [237]. Moreover, recent work involved the electrospinning of polycaprolactone (PCL) and polylactic-co-glycolic acid (PLGA) to generate stacked nanofibrous meshes as tissue-engineered 3D scaffolds revealing improved cell migration (Fig. 15h) [238].

The ultimate goal is to exploit these biofabrication techniques and develop a fully-functional, artificially-engineered tissue or organ, which could potentially address the alarming global shortage of transplantable organs, reduce the reliance on animal models for in vivo testing [239], and provide a realistic 3D organ platform for surgical simulation and pre-operative planning. Prof. Shu’s group envisages that the 3D bioprinted, organ-like surgical phantoms will play a key role towards the development of a digital twin-assisted surgery (DTAS) platform that can be applied to several types of surgery (open, minimally invasive, or robotic surgery).

## Conclusions and outlook

This review provides a non-exhaustive snapshot of biomanufacturing and biofabrication techniques being developed and utilised by research groups around the UK and Ireland. The resulting applications include improving the efficiency and accuracy of lab-grown biologically-based products, tissue engineering for a wide range of organ systems, and innovative biomaterial systems such as hydrogels, smart fibres, nanoclays, and bioactive implants. It would be impossible to include every biofabrication innovation in this review, a testament to the rapid evolution of this field. With this in mind, we have grouped research efforts into six research themes: bioprinting, drug delivery, biomaterials, tissue engineering, sustainability, and biohybrid. We highlight the work of selected research groups based on regional distribution and representation (Fig. 1). It is also important to note that while this review exclusively focuses on research groups based in the UK and Ireland, groups around the world are concurrently developing 3D biofabrication and biomanufacturing technologies similar to those discussed in this review [15, 16]. Through this geographical breakdown of biofabrication research in Ireland and the UK, we hope to promote collaboration both across regions and internationally. For example, the UK has benefited hugely from national funding schemes and networks such as the UK Regenerative Medicine Platform (UKRMP) hubs and the UK Research and Innovations (UKRI) Technology Touching Life Networks. Although these initiatives ended in 2022, they identified biofabrication as a key element to realizing the health and social benefits of bioproducts and regenerative medicine. Separately, the Engineering and Physical Sciences Research Council (EPSRC) of the UK and the Science Foundation Ireland (SFI) have an established agreement to welcome, encourage, and support research applications that cut across national boundaries involving collaborative teams led by researchers from the UK and Ireland [240].

In short, it is clear that biofabrication holds critical roles in facilitating engineering biology and sustainable biopharmaceutical and chemical manufacturing, meeting the national strategies for both the UK [241] and Ireland [242] respectively. There is an urgent need for new initiatives to be established in order to translate biofabrication in the UK and Ireland discussed in this review. In the near to medium term (3–5 years), pursuing engineering biology through biofabrication, which does not involve genetic modification, could expedite ethical compliance and public acceptance, thereby facilitating quicker return on investment in healthcare products and biotechnologies to be developed. Over the longer term (5–10 years), the integration of synthetic biology with biofabrication, alongside advances in tissue and cell engineering and scalable sustainable manufacturing, is expected to significantly enhance the value and safety of end products. This, in turn, may streamline regulatory processes due to heightened safety and efficacy. The success of these endeavours hinges on robust biofabrication and biomanufacturing sectors, ensuring swift adoption of new technologies for an ethical and sustainable bioeconomy as we transition through Industry 4.0 and into Industry 5.0.
